# New Pyrazolyl Thioureas Active against the *Staphylococcus* Genus

**DOI:** 10.3390/ph17030376

**Published:** 2024-03-15

**Authors:** Anna Maria Schito, Debora Caviglia, Susanna Penco, Andrea Spallarossa, Elena Cichero, Bruno Tasso, Chiara Brullo

**Affiliations:** 1 Department of Surgical Sciences and Integrated Diagnostics (DISC), University of Genoa, Viale Benedetto XV, 6, 16132 Genoa, Italy; amschito@unige.it (A.M.S.); debora.caviglia@edu.unige.it (D.C.); 2 Department of Pharmacy (DIFAR), Section of Medicinal Chemistry, University of Genoa, Viale Benedetto XV, 3, 16132 Genoa, Italy; andrea.spallarossa@unige.it (A.S.); elena.cichero@unige.it (E.C.); bruno.tasso@unige.it (B.T.); 3 Department of Experimental Medicine (DIMES), University of Genoa, Via L.B. Alberti, 2, 16132 Genoa, Italy

**Keywords:** pyrazoles, pyrazolyl thiourea, antibacterial activity, Gram-positive species, *Staphylococcus* genus, cystic fibrosis, in silico pharmacokinetic properties prediction

## Abstract

To meet the urgent need for new antibacterial molecules, a small library of pyrazolyl thioureas (PTUs) was designed, synthesized and tested against difficult-to-treat human pathogens. The prepared derivatives are characterized by a carboxyethyl functionality on C4 and different hydroxyalkyl chains on N1. Compounds **1a**–**o** were first evaluated against a large panel of Gram-positive and Gram-negative pathogens. In particular, the majority of PTUs proved to be active against different species of the *Staphylococcus* genus, with MIC values ranging from 32 to 128 µg/mL on methicillin-resistant *Staphylococcus* strains, often responsible for severe pulmonary disease in cystic fibrosis patients. Time-killing experiments were also performed for the most active compounds, evidencing a bacteriostatic mechanism of action. For most active derivatives, cytotoxicity was evaluated in Vero cells, and at the tested concentrations and at the experimental exposure time of 24 h, none of the compounds analysed showed significant toxicity. In addition, favourable drug-like, pharmacokinetic and toxicity properties were predicted for all new synthesized derivatives. Overall, the collected data confirmed the PTU scaffold as a promising chemotype for the development of novel antibacterial agents active against Gram-positive multi-resistant strains frequently isolated from cystic fibrosis patients.

## 1. Introduction

The World Health Organization (WHO) estimates that there are 700,000 casualties per year worldwide due to drug-resistant infections with a projection of 10 million deaths by 2050 and a general cost for the global economy up to USD 100 trillion. Unfortunately, the WHO also noted that in 2020, none of the 43 antibiotics in clinical use had fully solved the problem of drug resistance [1]. In this regard, the bacteria included in the ESKAPE group (*Enterococcus faecium*, *Staphylococcus aureus*, *Klebsiella pneumonia*, *Acinetobacter baumannii*, *Pseudomonas aeruginosa*, Enterobacter species) have been recognized by the Infectious Diseases Society of America (IDSA) as the most dangerous pathogens, due to their remarkable resistance to the most common conventional antibiotics. Additional concerns derived from the isolation of strains resistant to vancomycin and linezolid, considered the last line of defence against Gram-positive bacterial infections [2,3,4]. The clinical management of infections caused by these exceptional pathogens is often complex and problematic, especially in patients hospitalized or suffering from concomitant diseases.

In this regard, cystic fibrosis (CF) is a genetic disorder associated with the production of sticky and thick mucus that accumulates in different organs, including the lungs. This condition facilitates the adhesion and growth of pathogens in the respiratory epithelium and exposes CF patients to respiratory infections, mainly caused by methicillin-resistant *Staphylococcus aureus* (MRSA). Noteworthy, chronic lung infections with this Gram-positive multi-resistant pathogen have been associated with more severe pulmonary disease and increase the decline in lung function in CF patients [5,6]. Clinical studies on CF patients have shown that the incidence of methicillin-sensitive *S. aureus* (MSSA) is significantly lower than that of MRSA [7,8,9]. 

**Figure 1:**
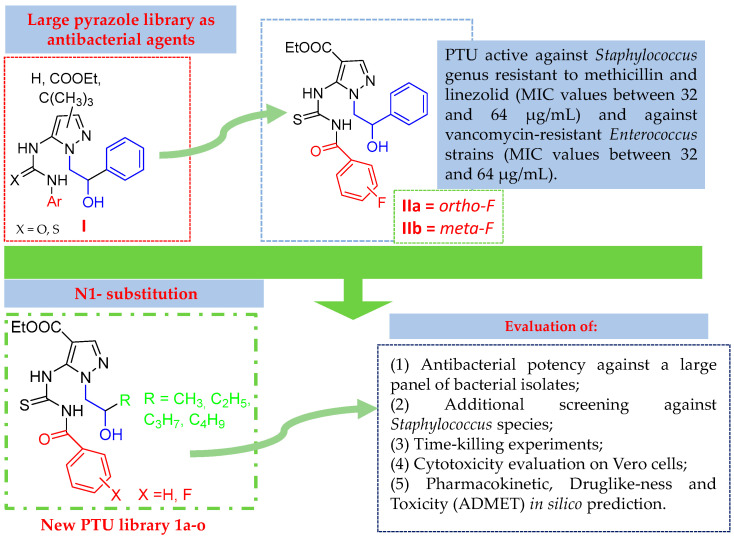
Structure of previous pyrazoles **I** and **II** and workflow of the applied strategies leading to the design of a novel small PTU library (compounds **1a**–**o**).

The pyrazole scaffold represents a privileged substructure in medicinal chemistry research and a number of pyrazole derivatives have been evaluated as effective compounds in different therapeutic areas [10,11,12,13,14,15,16], including infectious diseases [17,18] and also against MRSA [19]. In previous studies, pyrazole derivatives **I** (Figure 1) showed significative antibacterial activity against Gram-positive antibiotic-resistant strains [20]. The prepared molecules were characterized by a N1-hydroxy-2-phenylethyl chain, a carboxyethyl or *ter*t-butyl substituents on C3 or C4 and a (thio)ureido moiety at position 5 of the pyrazole ring. The structure activity relationships (SARs) evidenced that a carboxyethyl group on C4 and a substituted thioureido function on C5 are key structural determinants for the antimicrobial activity of this series (derivatives **II**, Figure 1). Pyrazolyl thioureas (PTUs) **II** resulted inactive against all tested Gram-negative species but showed a good antibacterial potency against strains of the *Staphylococcus* genus resistant to methicillin and linezolid (MIC values between 32 and 64 µg/mL) and also against vancomycin-resistant *Enterococcus* strains (MIC values between 32 and 64 µg/mL).

The subject of this work is the design and synthesis of a novel small library of PTU molecules (compounds **1a**–**o**) to confirm if this scaffold is a pharmaceutically relevant chemotype for the development of novel antibacterial agents, particularly active against Gram-positive species. 

## 2. Results and Discussion

The novel pyrazole small library of PTUs (compounds **1a**–**o**, Table 1) are characterized by: (1) a carboxyethyl function on C4 as previous derivatives **II**, (2) an unsubstituted or fluoro-substituted benzoyl thiourea segment on C5 and (3) various N1 hydroxyalkyl chains with different length, replacing the 2-hydroxy-2-phenylethyl chain of **I** and **II**. In detail, hydroxypropyl (compounds **1a**–**d**), hydroxybutyl (compounds **1e**–**h**), hydroxypentyl (compounds **1i**–**l**) and finally hydroxyhexyl (compounds **1m**–**o**) chains have been inserted on the N1 position of the pyrazole scaffold. 

Novel PTU library **1** was evaluated for their antibacterial activity against several Gram-positive and Gram-negative species, using oxacillin as the reference compound. Time-killing experiments on MRSA strains and cytotoxicity evaluation on Vero cells were also performed for the most active compounds. Additionally, in silico prediction of pharmacokinetic properties, drug-likeness and toxicity (ADMET) of all novel pyrazole compounds was performed.

### 2.1. Chemistry

**Scheme 1:**
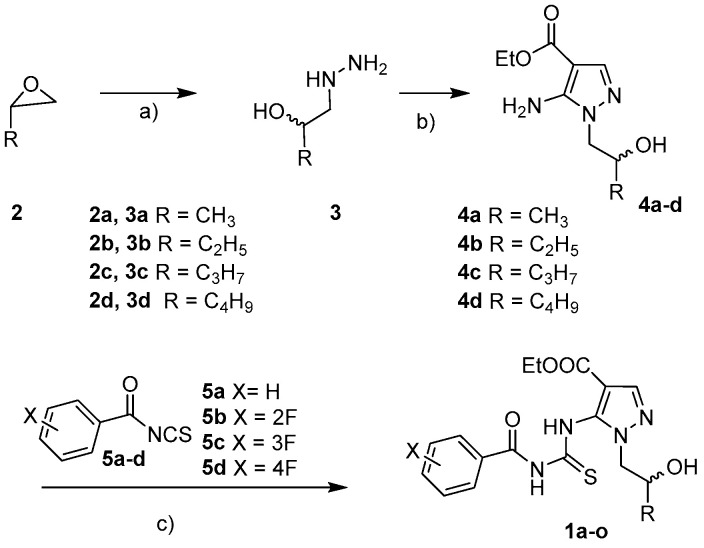
Synthesis of compounds **1a**–**o**. *Reagents and conditions*: (**a**) hydrazine monohydrate, 90–95 °C, 15 min–1 h 79–82%; (**b**) ethyl ethoxymethylene cyanoacetate, toluene, 70–80 °C, 8 h (**4a**) or abs. EtOH, 70–80 °C, 8 h, 61–72% (**4b**–**d**); (**c**) suitable benzoyl isothiocyanates **5a**–**d**, an. THF, reflux, 12 h, 35–94%.

Novel pyrazole library was obtained following a consolidate procedure [20], following Scheme 1. Briefly, the condensation of the commercially available oxiranes **2a**–**d** with hydrazine monohydrate led to the corresponding hydrazino-ethanols **3a**–**d,** that were reacted with ethyl ethoxymethylene cyanoacetate in anhydrous toluene (**3a**) or absolute ethanol (**3b**–**d**) at 70–80 °C to give the suitable pyrazole intermediates **4a**–**d** as yellow solids [21]. Finally, the thiourea moiety was introduced via a one-pot reaction in anhydrous THF for 12 h between the 5-amino-pyrazoles **4a**–**d** and the proper benzoyl isothiocyanate **5a**–**d**, commercially available or prepared according to the literature method [22]. Derivatives **1** were obtained in yields ranging from 35% to 94% (Table 1) as yellow oils or crystalline white solids.

### 2.2. Antibacterial Activity

The antibacterial potency of PTU **1a**–**o** was evaluated against a panel of fifteen bacterial isolates (Table 2), representative of clinically relevant Gram-positive (eleven strains) and Gram-negative (four strains) species including four *S. aureus* strains (MRSA), three *S. epidermidis* isolates (two MRSE and one resistant to methicillin and linezolid), two *E. faecalis* strains (one vancomycin-sensitive and one vancomycin resistant, VRE), two *E. faecium* isolates (one vancomycin-sensitive and one VRE), two *E. coli* isolates resistant to carbapenem (one was a New Delhi metallo-β-lactamase (NDM)-producing isolate) and two *P. aeruginosa* (multidrug-resistant isolates, MDR) strains.

All tested compounds resulted inactive (MIC > 128 µg/mL) against the Gram-negative and *Enterococcus* genus. Conversely, the majority of derivatives (10 out of 15) showed a widespread activity against the most clinically relevant *Staphylococcus* species (i.e., *S. aureus* MRSA and *S. epidermidis* MRSE), with MIC values lower in some cases than those of oxacillin, used as a reference compound (32–128 mg/mL against 128–512 mg/mL). Interestingly, PTUs **1i** and **1m** were identified as the most active derivatives of the series because they showed MIC values in a close range (32–64 µg/mL) against seven of the considered Gram-positive isolates. Also compounds **1d**, **1e**, **1k** and **1n** resulted active against six Gram-positive strains. 

To further define the antibacterial properties of the series, PTUs **1a**,**d**,**e**,**h**,**i**,**m** were selected as representative examples of differently N1 substituted pyrazoles (**1a** and **1d**, R = Me; **1e** and **1h**, R = Et; **1i**, R = *n*Pr; **1m**, R = *n*Bu) and tested against additional *Staphylococcus* species for a total of 14 isolates (Table 3). All analysed compounds proved to be ineffective against *S. saprophyticus, S. warneri* and *S. simulans,* but showed relevant antibacterial activity against *S. lugdunensis* and S*. auricularis* species (MIC value range = 16–32 µg/mL). Moreover, derivative **1d** specifically inhibited the proliferation of methicillin-resistant *S. capitis 71* strain (MIC = 64 µg/mL), whereas compound **1h** affected the growth of *S. hominis 124*, without influencing the *S. hominis 125*. Finally, derivatives **1d**,**e**,**h**,**i** showed similar activity against *S. haemoliticus 115* isolate, resulting ineffective against the other two considered *S. haemoliticus* strains. Regarding *S. auricularis*, compounds **1e**,**i**,**m** resulted the most potent, displaying MIC values of 16 µg/mL.

**Figure 2:**
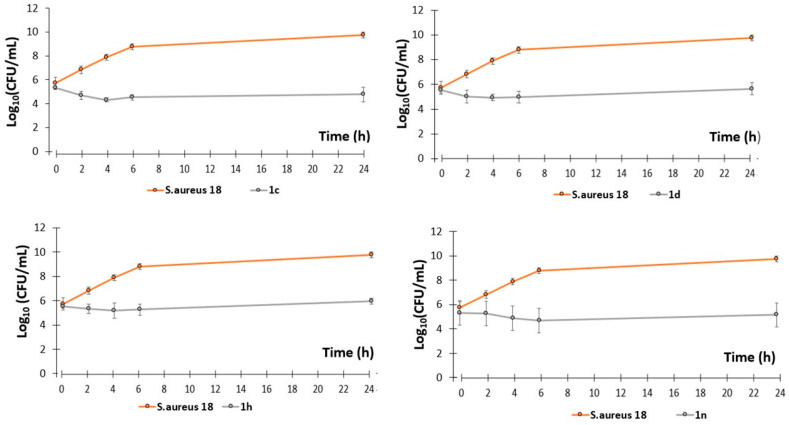
Time-killing curves performed with compounds **1c**, **1d**, **1n**, **1h** at a concentration of 4× MIC on *S. aureus* 18 (MRSA). Error bars represent standard deviations (n = 3) of the mean values. Similar results were obtained for *S. aureus* strains 17, 187 and 195.

To investigate whether PTUs act as bacteriostatic or bactericidal, time-killing experiments were carried out on MRSA isolates, very relevant in the daily clinical practice of CF patients. Compounds **1c**,**d**,**n**,**h** were selected as representative examples of the chemical diversity of the series and tested against four different MRSA strains (i.e., *S. aureus* 17, 18, 187 and 195). The experiments were carried out at concentrations four times the MIC values. As exemplified in Figure 2, the four tested compounds proved to act as a bacteriostatic agent, because they were all able to maintain virtually unchanged (10^5^ CFU/mL) the concentration of the initial bacterial inocula for all 24 h of this study. Similar trends were obtained for all the analysed MRSA strains.

### 2.3. Cytotoxicity Evaluation

**Figure 3:**
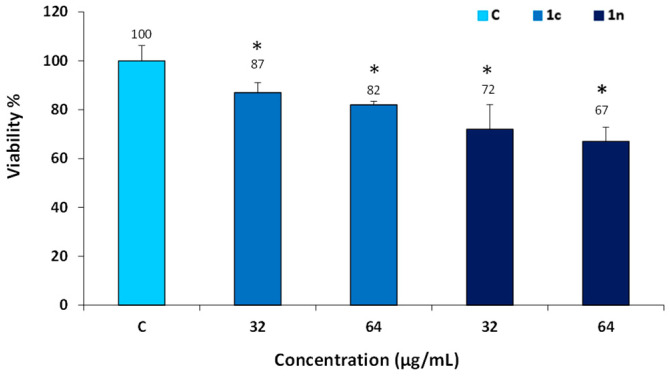
Cytotoxicity of compounds **1c** (in light blue) and **1n** (in blue) performed at concentrations 32 and 64 μg/mL on Vero cells. Data, expressed as percentage of viability versus untreated cultures and extrapolated by MTT assay, are the means ± SD of three separate experiments performed in triplicate. * = *p* < 0.01 versus untreated cultures (ANOVA and Dunnett test). At a concentration of 32 µg/mL, tested compounds showed the highest viability rates. According to ECVAM guidelines, compounds **1c** and **1n** can therefore be considered low toxic (at 32 µg/mL) and moderately toxic (at 64 µg/mL).

To verify if PTUs here reported are characterized by a cytotoxicity activity, selected compounds **1c** (R = methyl) and **1n** (R = butyl), chosen among the most active ones and as representative examples of the chemical diversity of the series, were tested on Vero cells at the most representative MIC values obtained (32 µg/mL and 64 µg/mL for both compounds, Figure 3). 

### 2.4. Pharmacokinetic Properties, Drug-Likeness and Toxicity (ADMET) Prediction 

To further characterize the pharmaceutical potentials of PTUs **1**, the drug-likeness and pharmacokinetic properties of the series were calculated using the SwissADME program [23]. Derivatives **IIa,b** (Figure 1) were used as reference molecules (Table 4). Collectively, this in silico evaluation predicted for PTUs **1** favourable physiochemical and DMPK properties that, in some case, would result better than those of previously described compounds **II**.

In detail, the replacement of the **II** phenyl group with aliphatic, linear chains (namely, methyl, ethyl, propyl or butyl R substituents, Table 1) led to an increase in the Csp_3_ fraction with an improvement of the predicted bioavailability. Respect to previous **II**, in most cases the number of rotatable bonds of H bond acceptors and H bond donors are the same of previous **II**. Moreover, **1** and **II** would display the similar polarity, as indicated by the topological polar surface area (TPSA) descriptor (137.57 Å^2^). This descriptor has proved to be indispensable for predicting the permeability of a molecule towards biological membranes. It has in fact been demonstrated that when the TPSA value is greater than 140 Å^2^, the molecules have difficulty permeating the barriers; on the contrary, when it is less than 140 Å^2^, the passage through the lipophilic barriers is easier. 

The different N1 substituents of the pyrazole ring would also affect the lipophilicity of the compounds, being the logPvalues within the desired range (logP between −0.7 and +5.0) for all analysed PTUs. Except for **1m**–**o** (N1 hydroxyhexyl derivatives), all compounds were predicted to be water soluble rather than moderately soluble as compounds **II**. 

Compounds **1a**–**i,1m** would be highly absorbed in the gastrointestinal tract, whereas derivatives **1j**–**l** and **1n**–**o**, characterized by a fluoro-substituted phenyl ring on thioureido function and a more embedded chain on N1 (hydroxypentyl and hydroxyexyl) would be poorly absorbed. As derivatives **II**, the novel compounds would not be able to pass the blood–brain barrier (BBB) and enter in the central nervous system. Furthermore, unlike the previous **II,** derivatives **1** would be substrates of the P-gp efflux pump. 

The CYP inhibition properties of compounds **1** would be different from that predicted for derivatives **II**. Thus, 1A2 and 2D6 isoforms would not be affected by PTUs **1,** while 2C19, 2C9 and 3A4 enzymes would be inhibited by derivatives **1e**–**o**.

As derivatives **II**, no violations of the Lipinski rules have been identified for PTUs **1** that would not display any pan-assay interference compounds (PAINS) alerts. According to the Brenk filters, the thiourea thiocarbonyl group on the C5 position was spotted as a problematic fragment [24]. 

**Table 4:** Predicted pharmacokinetics and drug-like properties of compounds **1a**–**o** in comparison with previously synthesized derivatives **IIa,b**.

	1a	1b–d	1e	1f–h	1i	1j–l	1m	1n–o	IIa,b
**Physicochemical Property**									
MW (g/mol)	376.43	394.42	390.46	408.45	404.48	422.47	418.51	436.50	456.49
Fraction Csp^3^	0.29	0.29	0.33	0.33	0.37	0.37	0.40	0.40	0.18
Rotatable bonds	10	10	11	11	12	12	13	13	11
H-bond acceptors	5	6	5	6	5	6	5	6	6
H-bond donors	3	3	3	3	3	3	3	3	3
TPSA ^a^ (Å^2^)	137.57	137.57	137.57	137.57	137.57	137.57	137.57	137.57	137.57
**Lipophilicity**									
LogP ^b^	2.06	2.16	2.59	2.69	2.95	3.05	3.49	3.59	3.26
**Water solubility**									
Solubility (mg/mL) ^c^	0.282	0.203	0.133	0.095	0.080	0.057	0.037	0.026	0.018
Solubility class ^d^	S	S	S	S	S	S	MS	MS	MS
**Pharmacokinetics**									
GI absorption	high	high	high	high	high	low	high	low	low
BBB permeant	no	no	no	no	no	no	no	no	no
P-gp substrate	yes	yes	yes	yes	yes	yes	yes	yes	no
CYP1A2 inhibitor	no	no	no	no	no	no	no	no	no
CYP2C19 inhibitor	no	no	yes	yes	yes	yes	yes	yes	yes
CYP2C9 inhibitor	no	no	yes	yes	yes	yes	yes	yes	yes
CYP2D6 inhibitor	no	no	no	no	no	no	no	no	yes
CYP3A4 inhibitor	no	no	yes	yes	yes	yes	yes	yes	yes
**Drug-likeness**									
Lipinski violations	0	0	0	0	0	0	0	0	0
**Medicinal chemistry**									
PAINS alerts	0	0	0	0	0	0	0	0	0
Brenk alerts	1	1	1	1	1	1	1	1	1

^a^ Topological polar surface area. ^b^ Predicted according to the XLOGP3 program. ^c^ Values predicted by the ESOL method [25]. ^d^ S = soluble; MS = moderately soluble.

Collectively, this in silico evaluation predicted for this new PTU library good physicochemical, lipophilicity and water solubility properties; in some cases, better than previous **II**.

In addition, also predicted acute toxicity (lethal dose, LD_50_) for rats after oral administration (Table 5) was calculated using the Advanced Chemistry Development (ACD) Percepta platform (ACD/Percepta Platform. Advanced Chemistry Development, Inc.; Toronto, ON, Canada: 2015). 

Reliability index values are shown as R.I. (values higher than 0.30 are ranked as reliable by the software). The software prediction is performed based on the software implemented training libraries, which include experimentally determined pharmacokinetic and safety properties for different series of compounds.

Notably, the newly developed compounds **1a**–**o** were predicted to have LD_50_ values in the 1600–3300 mg/kg range. 

## 3. Materials and Methods

### 3.1. Chemistry

#### 3.1.1. General Information

All solvents and reagents were purchased from Chiminord s.r.l. (Milan, Italy) and Merk (Aldrich Chemical, Milan, Italy). Solvents were reagent grade. All commercial reagents were used without further purification. For thin-layer chromatography (TLC), aluminium-backed silica gel plates (Merck DC-Alufolien Kieselgel 60 F254, Darmstad, Germany) were used. For chromatography, Merck silica gel, 230–400 mesh, was used. Flash chromatography was performed using the Isolera One instrument (Biotage, Uppsala, Sweden) using a silica gel column. Melting points were not “corrected” and were obtained with a Buchi M-560 instrument (Buchi instruments, Flawil, Switzerland). NMR spectra were recorded on a JEOL JNM ECZ-400S/L1 (400 MHz, Tokyo, Japan). 

Elemental analysis was determined with an elemental analyser EA 1110 (Fison-Instruments, Milan, Italy); compounds have been considered pure when the difference between calculated and found values is ± 0.4 (Table S1 in Supporting Material). Hydrazines **3** and pyrazole intermediates **4a**–**c** were prepared according to the already published procedures [21,26]. Benzoyl isothiocyanates **5b**–**d** were prepared according to the literature method [20,22].

#### 3.1.2. Synthesis of Ethyl 5-amino-1-(2-hydroxyhexyl)-1*H*-pyrazole-4-carboxylate **4d**

A mixture of ethyl ethoxymethylene acetate (3.38 g, 20 mmol) and 2-hydrazinohexane-2-ol **3d** (2.64 g, 20 mmol) [26] in absolute ethanol (40 mL) was heated at 70–80 °C for 8 h. After cooling at room temperature, the mixture was evaporated under vacuum; 6N HCl (30 mL) was added and the acid solution was washed with diethyl ether (20 mL), then alkalized with 4M NaOH to obtain a yellow solid that was filtered and recrystallized from a mixture of diethyl ether/ligroin (b.p. 40–60 °C) (1:1). 

Mp: 103–105 °C. Yield: 57%. ^1^H-NMR (400 MHz, DMSO-d_6_): δ 0.80 (t, J = 7.0, 3H, CH_3_), 1.07–1.34 (m, 9H, CH_3_ + 3CH_2_), 3.73–3.82 (m, 3H, *CH*OH + NCH_2_), 4.11 (q, J = 7.0, 2H, CH_2_O), 4.91 (d, 1H, OH, exchangeable with D_2_O), 6.05 (br s, 2H, NH_2_ exchangeable with D_2_O), 7.41 (s, 1H, H-3). ^13^C-NMR (101 MHz, DMSO-d_6_): δ 160.71, 149.11, 143.44, 98.61, 69.38, 55.67, 53.06, 31.80, 27.42, 23.44, 16.27. Anal. (C_12_H_21_N_3_O_3_) calcd for C, H, N.

#### 3.1.3. General Synthesis of 5-Thioureido Pyrazoles **1a**–**o**

The proper 5-amino-pyrazoles **4a**–**d** (1 mmol) and the suitable benzoyl isothiocyanate **5a**–**d** (1 mmol), commercially available or previously prepared modifying the literature method [22], in anhydrous THF (10 mL) was refluxed for 12 h. After cooling to room temperature, the solution was concentrated under reduced pressure and the crude was dissolved in ethyl acetate (20 mL); the organic phase was washed with 6N HCl (10 mL), then with NaHCO_3_ saturated solution (10 mL) and water (10 mL), dried (MgSO_4_) and concentrated under reduced pressure. The crude was purified by flash chromatography using a mixture of diethyl ether/ligroin (b.p. 40–60 °C) 3/1 as the eluent.

*Ethyl 5-(3-benzoylthioureido)-1-(2-hydroxypropyl)-1H-pyrazole-4-carboxylate* **1a**. White solid. M.p.: 106–108 °C. Yield: 64%. ^1^H-NMR (400 MHz, DMSO-d_6_): δ 1.07 (d, J = 6.0, 3H, CH_3_), 1.18 (t, J = 7.0, 3H, CH_3_), 3.91–4.05 (m, 3H, *CH*OH + CH_2_N), 4.13 (q, J = 7.0, 2H, CH_2_O), 5.05 (d, J = 4.8, 1H, OH exchangeable with D_2_O), 7.37–8.03 (m, 6H, 5 Ar + H-3), 12.10 (br s, 1H, NH, exchangeable with D_2_O), 12.20 (br s, 1H, NH, exchangeable with D_2_O). ^13^C-NMR (101 MHz, DMSO-d_6_): δ 180.47, 165.88, 161.44, 140.22, 139.93, 134.94, 132.03, 128.50, 128.09, 103.44, 66.87, 60.12, 57.55, 20.94, 14.28. Anal. (C_17_H_20_N_4_O_4_S) calcd for C, H, N, S.

*Ethyl 5-(3-(2-fluorobenzoyl)thioureido)-1-(2-hydroxypropyl)-1H-pyrazole-4-carboxylate* **1b**. Yellow oil. Yield: 45%. ^1^H-NMR (400 MHz, DMSO-d_6_): δ 1.05 (d, J = 6.0, 3H, CH_3_), 1.18 (t, *J* = 7.0, 3H, CH_3_), 3.90–4.09 (m, 3H, *CH*OH + CH_2_N), 4.12 (q, J = 7.0, 2H, CH_2_O), 5.05 (br s, 1H, OH exchangeable with D_2_O), 7.29–7.87 (m, 4H, Ar), 7.89 (s, 1H, H-3), 11.89 (br s, 1H, NH, exchangeable with D_2_O), 12.16 (br s, 1H, NH, exchangeable with D_2_O). ^13^C-NMR (101 MHz, DMSO-d_6_): δ 176.97, 164.60, 161.87, 158.29, 140.22, 131.76, 129.99, 122.75, 122.22, 115.82, 104.44, 67.63, 59.28, 55.77, 23.81, 16.39. Anal. (C_17_H_19_N_4_O_4_SF) calcd for C, H, N, S.

*Ethyl 5-(3-(3-fluorobenzoyl)thioureido)-1-(2-hydroxypropyl)-1H-pyrazole-4-carboxylate* **1c**. Yellow oil. Yield: 35%. ^1^H-NMR (400 MHz, DMSO-d_6_): δ 1.07 (d, *J* = 6.0, 3H, CH_3_), 1.22 (t, J = 7.0, 3H, CH_3_), 3.90–4.04 (m, 3H, *CH*OH + CH_2_N), 4.13 (q, J = 7.0, 2H, CH_2_O), 5.05 (d, J = 4.8, 1H, OH exchangeable with D_2_O), 7.52–7.87 (m, 4H, Ar), 7.92 (s, 1H, H-3), 12.04 (br s, 1H, NH, exchangeable with D_2_O), 12.16 (br s, 1H, NH, exchangeable with D_2_O). ^13^C-NMR (101 MHz, DMSO-d_6_): δ 178.28, 165.68, 162.48, 160.01, 158.56, 140.22, 140.09, 139.93, 130.66, 122.97, 115.03, 114.47, 104.20, 68.20, 60.08, 57.55, 21.16, 13.60. Anal. (C_17_H_19_N_4_O_4_SF) calcd for C, H, N, S.

*Ethyl 5-(3-(4-fluorobenzoyl)thioureido)-1-(2-hydroxypropyl)-1H-pyrazole-4-carboxylate* **1d**. White solid. M.p.: 131–133 °C. Yield: 39%. ^1^H-NMR (400 MHz, DMSO-d_6_): δ 1.16 (t, *J* = 6.0, 3H, CH_3_), 1.42 (t, J = 7.0, 3H, CH_3_), 4.09–4.30 (m, 3H, *CH*OH + CH_2_N), 4.32 (q, J = 7.0, 2H, CH_2_O), 5.05 (br s, 1H, OH exchangeable with D_2_O), 7.34–7.71 (m, 4H, Ar), 7.84 (s, 1H, H-3), 11.86 (br s, 1H, NH, exchangeable with D_2_O), 11.98 (br s, 1H, NH, exchangeable with D_2_O). ^13^C-NMR (101 MHz, DMSO-d_6_): δ 175.35, 166.01, 163.54, 161.44, 139.81, 130.17, 129.67, 115.99, 101.92, 68.20, 60.12, 57.55, 16.27, 9.97. Anal. (C_17_H_19_N_4_O_4_SF) calcd for C, H, N, S.

*Ethyl 5-(3-benzoylthioureido)-1-(2-hydroxybutyl)-1H-pyrazole-4-carboxylate* **1e**. Yellow oil. Yield: 55%. ^1^H-NMR (400 MHz, DMSO-d_6_): δ 0.88 (t, *J* = 6.0, 3H, CH_3_), 1.18 (t, *J* = 7.0, 3H, CH_3_), 1.22–1.47 (m, 2H, CH_2_), 3.72–4.04 (m, 3H, *CH*OH + CH_2_N), 4.14 (q, J = 7.0, 2H, CH_2_O), 5.02 (d, J = 4.8, 1H, OH exchangeable with D_2_O), 7.45–8.03 (m, 6H, 5Ar + H-3), 12.10 (br s, 1H, NH, exchangeable with D_2_O), 12.20 (br s, 1H, NH, exchangeable with D_2_O). ^13^C -NMR (101 MHz, DMSO-d_6_): δ 174.97, 167.07, 158.96, 141.86, 140.22, 136.90, 132.41, 128.50, 128.09, 101.68, 74.10, 60.95, 53.62, 30.22, 16.59, 7.13. Anal. (C_18_H_22_N_4_O_4_S) calcd for C, H, N, S.

*Ethyl 5-(3-(2-fluorobenzoyl)thioureido)-1-(2-hydroxybutyl)-1H-pyrazole-4-carboxylate* **1f**. Yellow oil. Yield: 41%. ^1^H-NMR (400 MHz, DMSO-d_6_): δ 0.88 (t, *J* = 6.0, 3H, CH_3_), 1.03–1.46 (m, 5H, CH_3_ + CH_2_), 3.74–4.04 (m, 3H, *CH*OH + CH_2_N), 4.14 (q, J = 7.0, 2H, CH_2_O), 5.05 (d, J = 4.8, 1H, OH exchangeable with D_2_O), 7.26–7.90 (m, 4H, Ar), 7.92 (s, 1H, H-3), 11.92 (br s, 1H, NH, exchangeable with D_2_O), 12.15 (br s, 1H, NH, exchangeable with D_2_O). ^13^C-NMR (101 MHz, DMSO-d_6_): δ 176.97, 164.60, 161.47, 158.99, 140.22, 132.22, 129.99, 125.06, 123.62, 116.34, 103.44, 71.50, 60.12, 54.73, 27.50, 14.28, 9.79. Anal. (C_18_H_21_N_4_O_4_SF) calcd for C, H, N, S.

*Ethyl 5-(3-(3-fluorobenzoyl)thioureido)-1-(2-hydroxybutyl)-1H-pyrazole-4-carboxylate* **1g**. Yellow oil. Yield: 40%. ^1^H-NMR (400 MHz, DMSO-d_6_): δ 0.88 (t, J = 6.0, 3H, CH_3_), 1.16–1.43 (m, 5H, CH_3_ + CH_2_), 3.74–4.04 (m, 3H, *CH*OH + CH_2_N), 4.14 (q, J = 7.0, 2H, CH_2_O), 5.02 (d, J = 4.8, 1H, OH exchangeable with D_2_O), 7.38–7.89 (m, 4H, Ar), 7.92 (s, 1H, H-3), 12.07 (br s, 1H, NH, exchangeable with D_2_O), 12.16 (br s, 1H, NH, exchangeable with D_2_O). ^13^C-NMR (101 MHz, DMSO-d_6_): δ 177.08, 166.55, 162.79, 161.44, 160.33, 140.22, 137.37, 130.66, 123.42, 118.99, 115.19, 105.38, 74.10, 63.07, 54.64, 30.54, 15.56, 11.31. Anal. (C_18_H_21_N_4_O_4_SF) calcd for C, H, N, S.

*Ethyl 5-(3-(4-fluorobenzoyl)thioureido)-1-(2-hydroxybutyl)-1H-pyrazole-4-carboxylate* **1h**. White solid. M.p.: 120–122 °C. Yield: 50%. ^1^H-NMR (400 MHz, DMSO-d_6_): δ 1.21 (t, *J* = 6.0, 3H, CH_3_), 1.60 (t, J = 7.0, 3H, CH_3_), 1.63–1.79 (m, 2H, *CH_2_*CH_3_), 4.13–4.28 (m, 3H, *CH*OH + CH_2_N), 4.52 (q, J = 7.0, 2H, CH_2_O), 5.05 (d, J = 4.8, 1H, OH exchangeable with D_2_O), 7.33–7.38 and 7.65–7.72 (2m, 4H, Ar), 7.88 (s, 1H, H-3), 11.72 (br s, 1H, NH, exchangeable with D_2_O), 11.82 (br s, 1H, NH, exchangeable with D_2_O). ^13^C-NMR (101 MHz, DMSO-d_6_): δ 177.08, 166.01, 165.26, 163.54, 159.75, 139.50, 130.17, 118.10, 101.44, 71.50, 54.17, 47.55, 24.23, 10.29, 5.80. Anal. (C_18_H_21_N_4_O_4_SF) calcd for C, H, N, S.

*Ethyl 5-(3-benzoylthioureido)-1-(2-hydroxypentyl)-1H-pyrazole-4-carboxylate* **1i**. White solid. M.p.: 126–128 C. Yield: 55%. ^1^H-NMR (400 MHz, DMSO-d_6_): δ 0.83 (t, *J* = 6.0, 3H, CH_3_), 1.18 (t, *J* = 7.0, 3H, CH_3_), 1.25–1.43 (m, 4H, 2CH_2_), 3.81–4.03 (m, 3H, *CH*OH + CH_2_N), 4.14 (q, J = 7.0, 2H, CH_2_O), 5.00 (d, J = 4.8, 1H, OH exchangeable with D_2_O), 7.55–8.02 (m, 6H, 5Ar + H-3), 12.10 (br s, 1H, NH, exchangeable with D_2_O), 12.19 (br s, 1H, NH, exchangeable with D_2_O). ^13^C-NMR (101 MHz, DMSO-d_6_): δ 177.06, 165.63, 161.55, 140.22, 140.08, 135.30, 132.14, 128.66, 128.50, 128.09, 128.01, 103.44, 70.17, 60.12, 55.34, 37.09, 18.44, 14.28, 14.12. Anal. (C_19_H_24_N_4_O_4_S) calcd for C, H, N, S.

*Ethyl 5-(3-(2-fluorobenzoyl)thioureido)-1-(2-hydroxypentyl)-1H-pyrazole-4-carboxylate* **1j**. Yellow oil. Yield: 36%. ^1^H-NMR (400 MHz, DMSO-d_6_): δ 0.83 (t, *J* = 6.0, 3H, CH_3_), 1.15–1.42 (m, 7H, CH_3_ +2CH_2_), 3.82–4.05 (m, 3H, *CH*OH + CH_2_N), 4.15 (q, J = 7.0, 2H, CH_2_O), 5.02 (d, J = 4.8, 1H, OH exchangeable with D_2_O), 7.34–7.90 (m, 4H, Ar), 7.92 (s, 1H, H-3), 11.92 (br s, 1H, NH, exchangeable with D_2_O), 12.10 (br s, 1H, NH, exchangeable with D_2_O). ^13^C-NMR (101 MHz, DMSO-d_6_): δ 175.74, 164.60, 161.47, 158.99, 140.22, 132.22, 132.14, 125.06, 123.62, 116.34, 106.17, 73.08, 60.12, 55.34, 37.09, 20.30, 11.86. Anal. (C_19_H_23_N_4_O_4_SF) calcd for C, H, N, S.

*Ethyl 5-(3-(3-fluorobenzoyl)thioureido)-1-(2-hydroxypentyl)-1H-pyrazole-4-carboxylate* **1k**. Yellow oil. Yield: 51%. ^1^H-NMR (400 MHz, DMSO-d_6_): δ 0.84 (t, *J* = 6.0, 3H, CH_3_), 1.20–1.42 (m, 7H, CH_3_ +2CH_2_), 3.82–4.02 (m, 3H, *CH*OH + CH_2_N), 4.14 (q, J = 7.0, 2H, CH_2_O), 4.99 (br s, 1H, OH exchangeable with D_2_O), 7.52–7.87 (m, 4H, Ar), 7.91 (s, 1H, H-3), 12.11 (br s, 1H, NH, exchangeable with D_2_O). ^13^C-NMR (101 MHz, DMSO-d_6_): δ 178.12, 167.65, 162.79, 161.44, 160.33, 140.22, 135.37, 130.66, 123.42, 119.36, 103.44, 69.14, 59.92, 54.41, 35.50, 18.44, 14.12. Anal. (C_19_H_23_N_4_O_4_SF) calcd for C, H, N, S.

*Ethyl 5-(3-(4-fluorobenzoyl)thioureido)-1-(2-hydroxypentyl)-1H-pyrazole-4-carboxylate* **1l**. White solid. M.p.: 134–136 °C. Yield: 61%. ^1^H-NMR (400 MHz, DMSO-d_6_): δ 0.84 (t, *J* = 6.0, 3H, CH_3_), 1.10–1.40 (m, 7H, CH_3_ +2CH_2_), 3.82–3.99 (m, 3H, *CH*OH + CH_2_N), 4.13 (q, J = 7.0, 2H, CH_2_O), 5.00 (br s, 1H, OH exchangeable with D_2_O), 7.38–7.44-(m, 2H, Ar), 7.92 (s, 1H, H-3), 8.05–8.12 (m, 2H, Ar), 12.14 (br s, 1H, NH, exchangeable with D_2_O). ^13^C-NMR (101 MHz, DMSO-d_6_): δ 174.73, 166.01, 163.54, 161.44, 140.22, 130.26, 115.99, 105.38, 70.72, 60.71, 55.43, 37.09, 18.44, 13.99, 13.20. Anal. (C_19_H_23_N_4_O_4_SF) calcd for C, H, N, S.

*Ethyl 5-(3-benzoylthioureido)-1-(2-hydroxyhexyl)-1H-pyrazole-4-carboxylate* **1m**. White solid. M.p.: 108–110 °C. Yield: 43%. ^1^H-NMR (400 MHz, DMSO-d_6_): δ 0.80 (t, *J* = 6.0, 3H, CH_3_), 1.15 (t, *J* = 7.0, 3H, CH_3_), 1.20–1.38 (m, 6H, 3CH_2_), 3.76–4.07 (m, 3H, *CH*OH + CH_2_N), 4.12 (q, J = 7.0, 2H, CH_2_O), 4.96 (br s, 1H, OH exchangeable with D_2_O), 7.41–7.98 (m, 6H, 5Ar + H-3), 12.06 (br s, 1H, NH, exchangeable with D_2_O), 12.18 (br s, 1H, NH, exchangeable with D_2_O). ^13^C-NMR (101 MHz, DMSO-d_6_): δ 174.73, 166.52, 159.98, 140.22, 140.08, 137.13, 133.20, 128.50, 128.09, 127.68, 102.23, 71.74, 63.63, 57.32, 36.52, 25.26, 21.32, 14.46, 13.20. Anal. (C_20_H_26_N_4_O_4_S) calcd for C, H, N, S.

*Ethyl 5-(3-(3-fluorobenzoyl)thioureido)-1-(2-hydroxyhexyl)-1H-pyrazole-4-carboxylate* **1n**. Yellow oil. Yield: 47%. ^1^H-NMR (400 MHz, DMSO-d_6_): δ 0.85–0.96 (m, 6H, 2CH_3_), 1.27–1.31 (m, 6H, 3CH_2_), 3.95–4.25 (m, 3H, *CH*OH + CH_2_N), 4.30 (q, J = 7.0, 2H, CH_2_O), 7.35–7.92 (m, 4H, Ar), 7.98 (s, 1H, H-3), 9.49 (br s, 1H, NH, exchangeable with D_2_O), 12.09 (br s, 1H, NH, exchangeable with D_2_O). ^13^C-NMR (101 MHz, DMSO-d_6_): δ 174.95, 168.91, 162.24, 161.44, 159.77, 140.29, 136.15, 130.66, 123.42, 118.99, 114.95, 103.44, 70.23, 60.12, 55.30, 35.74, 27.42, 22.70, 14.28, 12.41. Anal. (C_20_H_25_N_4_O_4_SF) calcd for C, H, N, S.

*Ethyl 5-(3-(4-fluorobenzoyl)thioureido)-1-(2-hydroxyhexyl)-1H-pyrazole-4-carboxylate* **1o**. White solid. M.p.: 114–116 °C. Yield: 94%. ^1^H-NMR (400 MHz, DMSO-d_6_): δ 0.84 (t, *J* = 6.0, 3H, CH_3_), 1.07–1.39 (m, 9H, CH_3_ +3CH_2_), 3.80–4.03 (m, 3H, *CH*OH + CH_2_N), 4.13 (q, J = 7.0, 2H, CH_2_O), 5.00 (d, J = 4.8, 1H, OH exchangeable with D_2_O), 7.38–7.44 (m, 2H, Ar), 7.92 (s, 1H, H-3), 8.07–8.12 (m, 2H, Ar), 12.14 (br s, 1H, NH, exchangeable with D_2_O). ^13^C-NMR (101 MHz, DMSO-d_6_): δ 175.68, 167.91, 167.00, 165.45, 163.13, 139.81, 138.95, 132.11, 130.17, 117.86, 115.76, 102.23, 67.88, 59.53, 53.30, 33.61, 26.67, 22.26, 11.23, 10.92. Anal. (C_20_H_25_N_4_O_4_SF) calcd for C, H, N, S.

### 3.2. Microbiological Evaluation

#### 3.2.1. Bacterial Species Considered in This Study

A total of 29 clinical strains were used in this study, all belonging to a collection obtained from the School of Medicine and Pharmacy of University of Genoa (Italy), identified by VITEK^®^ 2 (Biomerieux, Firenze, Italy) or the matrix-assisted laser desorption/ionization time-of-flight (MALDI-TOF) mass spectrometric technique (Biomerieux, Firenze, Italy).

Particularly, fifteen strains were used to preliminary test the 15 new PTU derivatives, which were eleven Gram-positive strains (four MRSA *Staphylococcus aureus* strains), three*Staphylococcus epidermidis* isolates (two MRSE and one resistant to methicillin and linezolid), two *Enterococcus faecalis* strains (one vancomycin-sensitive and one vancomycin-resistant (VRE)), two *Enterococcus faecium* isolates (one vancomycin-sensitive and one (VRE)) and four Gram-negative isolates (two *Escherichia coli* isolates, one was a New Delhi metallo-β-lactamase (NDM)-producing isolate and both carbapenem resistant, and two *Pseudomonas aeruginosa* MDR isolates).

In addition, fourteen isolates of the *Staphylococcus* genus were also evaluated, including one *S. saprophyticus*, two *S. capitis* (one was resistant to methicillin), one *S. warneri* (resistant to methicillin), two *S. simulans* (both resistant to methicillin), two *S. lugdunensis* (one was resistant to methicillin), three *S. haemoliticus* (two were resistant to methicillin), two *S. hominis* (both resistant to methicillin) and one *S. auricularis* (resistant to methicillin). 

#### 3.2.2. Determination of the Minimal Inhibitory Concentrations (MICs) 

MIC values were determined following the microdilution procedures detailed by the European Committee on Antimicrobial Susceptibility Testing EUCAST [27], as also reported in our previous studies [20,28]. Briefly, serial two-fold dilutions in Mueller–Hinton (MH) broth (Merck, Darmstadt, Germany) of all the fifteen samples (dissolved in DMSO), ranging from 128 to 1 µg/mL, were used. DMSO was also tested as a control to verify the absence of antibacterial activity of the solvent used for the experiments. Cultures of all the selected bacteria, after overnight incubation, were diluted to yield a standardized inoculum of 1.5 × 10^8^ CFU/mL. Appropriate aliquots of each suspension were added to 96-well microplates containing dilutions of compounds to be tested to yield a final concentration of about 5 × 10^5^ cells/mL. After 24 h of incubation at 37 °C, the lowest concentration of sample that prevented visible growth was recorded as the MIC. All MICs were obtained at least in triplicate and results were expressed reporting the modal value; that is, the value that has been observed most frequently. In case of equivocal or not clear results, more than three determinations of MICs were carried out.

#### 3.2.3. Killing Curves

Killing curve assays for most interesting compounds (**1a**, **1e**, **1i** and **1m**) were performed on the four MRSA isolates selected for this study as previously reported [20,28].

A mid logarithmic phase bacterial culture was diluted in MH broth (10 mL) containing 4× MIC of all the compounds to give a final inoculum of 3.0 × 10^5^ CFU/mL. The same inoculum was added to MH broth, as a growth control. Tubes were incubated at 37 °C with constant shaking for 24 h. Samples of 0.20 mL from each tube were removed at 0, 2, 4, 6 and 24 h, diluted appropriately with a 0.9% sodium chloride solution to avoid carryover of compounds being tested, plated onto MH plates and incubated for 24 h at 37 °C. Growth controls were run in parallel. The percentage of surviving bacterial cells was determined for each sampling time by comparing colony counts with those of standard dilutions of the growth control. Results have been expressed as log_10_ of viable cell numbers (CFU/mL) of of surviving bacterial cells over a 24 h period. All time-kill curve experiments were performed in triplicate.

### 3.3. Maintenance of Cell Cultures

Vero cell line, isolated from kidney epithelia cells extracted from *Cercopithecus aethiops*, was certified by STR DNA profile analysis by Biological Bank, a Core Facility of the IRCCS San Martino University Hospital-IST National Institute for Cancer Research (Genoa, Italy). Vero cells were routinely cultured al 37 °C under 5% CO_2_ in a DMEM medium (Euroclone, Milan, Italy) plus 10% heat inactivated FBS serum (Euroclone, Milan, Italy). No antibiotic or anti-fungine solutions were added to the standard or experimental medium in order to avoid any potential interference of these drugs with the experimental conditions. The medium was changed every 2 to 3 days and cells were sub-cultured by TripLE^TM^ Express (Invitrogen, Life Technologies, Carlsbad, CA, USA) treatment when the original flask was approximately 75% confluent. All cell cultures were found to be mycoplasma-free during regular checks with the Reagent Set Mycoplasma Euroclone (Euroclone, Milan, Italy).

#### 3.3.1. Cell Viability Index

##### MTT Test

At the end of each experimental treatment, the cell viability was assessed by thiazolyl blue tetrazolium dye reduction assay (MTT) (Euroclone, Milan, Italy) [29]. The optical densities (OD) of the dissolved formazan crystals (for the MTT test) were determined spectrophotometrically at 570 nm.

A chemical compound was considered toxic if the cell viability was reduced by 15% compared to untreated cultures, according to the ECVAM’s guidelines testing any cytotoxic effects of the compounds and in parallel for measuring cell proliferation, according to manufacturer’s instructions.

## 4. Conclusions

The obtained results pointed at the PTU scaffold as a pharmaceutically relevant chemotype for the development of novel antibacterial agents active against Gram-positive species. In fact, novel PTUs here reported proved to be equally or more active that previous derivatives **I**. Compounds **1a**–**o** evidenced interesting activity towards Gram-positive resistant pathogen, often associated with severe pulmonary disease in CF patients [30,31,32,33,34]; in fact, several novel PTUs proved to be effective against different species of the *Staphylococcus* genus, with MIC values ranging from 32 to 128 µg/mL on MRSA and MRSE strains. In addition, time-killing experiments confirm the bacteriostatic actions of this class of compounds.

For all the synthesized compounds, favourable pharmacokinetic properties were calculated, evidencing for a major part of PTUs’ good ADMET properties. Finally, considering the preliminary cytotoxicity results obtained on Vero cells, it is reasonable to assume that the PTU library here reported have low toxicity.

**Figure 4:**
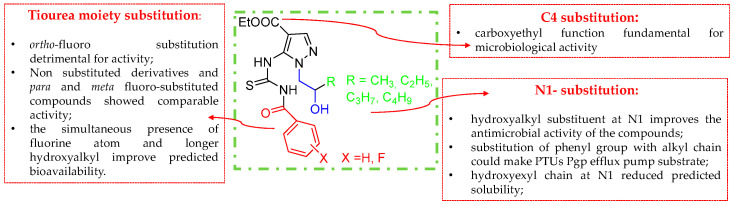
SAR considerations about PTUs here reported.

Collected data allow us to draw the following SAR considerations regarding N1 and thiourea moiety substitution (Figure 4): (1) the replacement of hydroxy-2-phenylethyl chain with hydroxyalkyl substituent at N1 improves the antimicrobial activity of the compounds, but make PTUs here reported P-gp efflux pump substrates; (2) the presence of the N1 hydroxyexyl chain at N1 reduced solubility (compounds **1m**–**o**); (3) the *ortho*-fluoro substitution of the thiourea benzoyl ring is detrimental for activity (as in derivatives **1b**,**f,j**), whereas *para* and *meta* fluoro-substituted compounds evidenced a potency comparable to their non-substituted analogues (**1a**,**e**,**i**,**m**); (4) the simultaneous presence of the fluorine atom and longer hydroxyalkyl chain (hydroxypentyl **1j**–**l** or hydroxyexyl **1n**,**1o**) increases the Csp3 fraction with an improvement of the predicted bioavailability; (5) the carboxyethyl function at C4 position resulted relevant for biological activity, as previously evidenced for derivatives **IIa,b**. 

Additional chemical modifications on the C4 and C3 pyrazole nucleus (as in previous derivatives **I** [20]) will be performed to extend the SAR consideration about the PTU chemotype.

As in our previous studies on pyrazole derivatives **I** and **II** [20], these collected results supported that, upon nano-formulation with proper polymer matrices, the new synthesized compounds could provide novel pyrazole-based drug delivery systems with enhanced and enlarged-spectrum of antibacterial activity, particularly against Gram-positive MRSA and MRSE species. 

In conclusion, PTUs here reported could represent a new starting point for the development of new antibacterial pyrazole-based agents [35,36,37,38] able to counteract strains resistant to common antibiotics and consequently a new therapeutic approach for CF patients [39,40,41]. Additional studies will be necessary to identify the mechanism of action of this class of molecules.

## Figures and Tables

**Table 1:** Chemical structure, melting point and reaction yield of novel PTU derivatives **1a**–**o**.

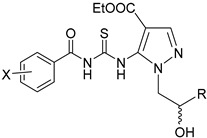				
Compound	R	X	Melting Point (°C)	Yield (%)
**1a**	CH_3_	H	106–108 °C	64%
**1b**	CH_3_	*o*-F	Yellow oil	45%
**1c**	CH_3_	*m*-F	Yellow oil	35%
**1d**	CH_3_	*p*-F	131–133 °C	39%
**1e**	C_2_H_5_	H	Yellow oil	55%
**1f**	C_2_H_5_	*o*-F	Yellow oil	41%
**1g**	C_2_H_5_	*m*-F	Yellow oil	40%
**1h**	C_2_H_5_	*p*-F	120–122 °C	50%
**1i**	C_3_H_7_	H	126–128 °C	55%
**1j**	C_3_H_7_	*o*-F	Yellow oil	36%
**1k**	C_3_H_7_	*m*-F	Yellow oil	51%
**1l**	C_3_H_7_	*p*-F	134–136 °C	61%
**1m**	C_4_H_9_	H	108–110 °C	43%
**1n**	C_4_H_9_	*m*-F	Yellow oil	47%
**1o**	C_4_H_9_	*p*-F	114–116 °C	94%

**Table 2:** MIC values (expressed as µg/mL) of PTU **1** and reference compound oxacillin (oxa) against bacteria of the Gram-positive and Gram-negative species, obtained from experiments carried out at least in triplicate. * denotes resistant to methicillin; ** denotes resistant to methicillin and linezolid; ^#^ denotes resistance to vancomycin; NDM: New Delhi metallo-beta-lactamase producer; MDR: multidrug-resistant strain; CR carbapenem resistant. In bold are evidenced the most active compounds.

	MIC (µg/mL)															
Strain	1a	1b	1c	1d	1e	1f	1g	1h	1i	1j	1k	1l	1m	1n	1o	oxa
**Gram-positive**																
*S. aureus* 17 *	128	>128	**64**	128	128	>128	**64**	128	**64**	>128	**64**	>128	**64**	**64**	>128	256
*S. aureus* 18 *	128	>128	**64**	**64**	**64**	>128	128	128	**64**	>128	**64**	>128	**64**	**64**	>128	512
*S. aureus* 187 *	**64**	>128	**32**	**32**	**64**	>128	**64**	**64**	**64**	>128	**32**	>128	**32**	**32**	>128	512
*S. aureus* 195 *	**64**	>128	**64**	**64**	**64**	>128	**64**	**64**	**64**	>128	**64**	>128	**32**	**64**	>128	256
*S. epidermidis* 22 *	**32**	>128	**64**	**32**	**32**	>128	>128	**64**	**32**	>128	**64**	>128	**32**	**64**	>128	256
*S. epidermidis* 171 *	**64**	>128	128	**32**	**64**	>128	128	**32**	**64**	>128	**64**	>128	**64**	**64**	>128	128
*S. epidermidis* 181 **	**64**	>128	64	**32**	**64**	>128	128	**32**	**64**	>128	128	>128	**64**	128	>128	256
*E. faecalis* 365 ^#^	>128	>128	>128	>128	>128	>128	>128	>128	>128	>128	>128	>128	>128	>128	>128	-
*E. faecalis* 28	>128	>128	>128	>128	>128	>128	>128	>128	>128	>128	>128	>128	>128	>128	>128	-
*E. faecium* 152 ^#^	>128	>128	>128	>128	>128	>128	>128	>128	>128	>128	>128	>128	>128	>128	>128	-
*E. faecium* 158	>128	>128	>128	>128	>128	>128	>128	>128	>128	>128	>128	>128	>128	>128	>128	-
**Gram-negative**																
*E. coli* 462 (*NDM*, *CR*)	>128	>128	>128	>128	>128	>128	>128	>128	>128	>128	>128	>128	>128	>128	>128	-
*E. coli* 475 (*CR*)	>128	>128	>128	>128	>128	>128	>128	>128	>128	>128	>128	>128	>128	>128	>128	-
*P. aeruginosa 1V MDR*	>128	>128	>128	>128	>128	>128	>128	>128	>128	>128	>128	>128	>128	>128	>128	-
*P. aeruginosa 6G MDR*	>128	>128	>128	>128	>128	>128	>128	>128	>128	>128	>128	>128	>128	>128	>128	-

**Table 3:** MIC values of some PTUs **1** and reference compound oxacillin (oxa) against other *Staphylococcus* species, obtained from experiments carried out at least in triplicate, expressed as µg/mL. * denotes resistant to methicillin. In bold are evidenced the most active compounds.

	MIC (µg/mL)						
Strains	1a	1d	1e	1h	1i	1m	oxa
*S. saprophyticus* 41	>128	>128	>128	>128	>128	>128	0.5
*S. capitis* 71 *	128	**64**	128	128	128	128	64
*S. capitis* 121	>128	128	>128	128	>128	>128	0.25
*S. warneri* 74 *	>128	>128	>128	>128	>128	>128	64
*S. simulans* 94 *	>128	>128	>128	>128	>128	>128	16
*S. simulans* 163 *	>128	>128	>128	>128	>128	>128	16
*S. lugdunensis* 96	**32**	**32**	**32**	**32**	**32**	**32**	1
*S. lugdunensis* 137 *	**32**	**32**	**16**	**32**	**16**	**32**	16
*S. haemoliticus* 115 *	128	**64**	**64**	**64**	**64**	128	64
*S. haemoliticus* 193 *	>128	128	128	128	>128	>128	16
*S. haemoliticus* 174	>128	>128	128	>128	>128	>128	0.25
*S. hominis* 124 *	128	128	128	**64**	128	>128	16
*S. hominis* 125 *	128	128	128	128	128	128	24
*S. auricularis* 136 *	**32**	**32**	**16**	**32**	**16**	**16**	16

**Table 5:** Predicted toxicity properties of compounds **1a**–**o**.

Cpd	LD_50_ (mg/kg)	Reliability (R.I.)
**1a**	2600	0.45
**1b–d**	1600	0.39
**1e**	2800	0.45
**1f**–**h**	1700	0.39
**1i**	3000	0.45
**1j**–**l**	1900	0.40
**1m**	3300	0.45
**1n**–**o**	2000	0.40

## Data Availability

Data are contained within the article.

## Supplementary Materials

The following supporting information can be downloaded at: https://www.mdpi.com/article/10.3390/ph17030376/s1, Elemental analysis, ^1^H NMR (400 MHz) and ^13^C NMR (101 MHz) of compounds **4d**, **1a**–**o** are reported.
