# Supplementary material for: New Pyrazolyl Thioureas Active against the Staphylococcus Genus
Source: Pharmaceuticals (Basel). 2024 Mar 15;17(3):376. doi: 10.3390/ph17030376 (PMC10974609; doi:10.3390/ph17030376)

New pyrazolyl thioureas active against the *Staphylococcus* genus

Anna Maria Schito [1], Debora Caviglia [1,2], Susanna Penco [3], Andrea Spallarossa [2], Elena Cichero [2], Bruno Tasso [2] and Chiara Brullo [2] *

1 Department of Surgical Sciences and Integrated Diagnostics (DISC), University of Genoa, Viale Benedetto XV, 6, 16132 Genoa, Italy; amschito@unige.it (A.M.S.), debora.caviglia@edu.unige.it (D.C)
2 Department of Pharmacy (DIFAR), Section of Medicinal Chemistry, University of Genoa, Viale Benedetto XV, 3, 16132 Genoa, Italy; andrea.spallarossa@unige.it (A.S.), bruno.tasso@unige.it (B.T.), elena.cichero@unige.it (E.C.)
3 Department of Experimental Medicine (DIMES), University of Genoa, Via L.B. Alberti, 2, 16132 Genoa, Italy; susanna.penco@unige.it (S.P.)
* Correspondence: chiara.brullo@unige.it

Supporting Material:

Table S1. Elemental analysis of compounds **1** and **4d.**
Figure S1:^{1}H NMR (400 MHz) of compound **1a**
Figure S2: ^{13}C NMR (101 MHz) of compound **1a.**
Figure S3:^{1}H NMR (400 MHz) of compound **1b.**
Figure S4: ^{13}C NMR (101 MHz) of compound **1b.**
Figure S5:^{1}H NMR (400 MHz) of compound **1c.**
Figure S6: ^{13}C NMR (101 MHz) of compound **1c.**
Figure S7:^{1}H NMR (400 MHz) of compound **1d.**
Figure S8: ^{13}C NMR (101 MHz) of compound **1d.**
Figure S9:^{1}H NMR (400 MHz) of compound **1e.**
Figure S10: ^{13}C NMR (101 MHz) of compound **1e.**
Figure S11:^{1}H NMR (400 MHz) of compound **1f.**
Figure S12: ^{13}C NMR (101 MHz) of compound **1f.**
Figure S13:^{1}H NMR (400 MHz) of compound **1g.**
Figure S14: ^{13}C NMR (101 MHz) of compound **1g.**
Figure S15:^{1}H NMR (400 MHz) of compound **1h.**
Figure S16: ^{13}C NMR (101 MHz) of compound **1h.**
Figure S17:^{1}H NMR (400 MHz) of compound **1i.**
Figure S18: ^{13}C NMR (101 MHz) of compound **1i.**
Figure S19:^{1}H NMR (400 MHz) of compound **1j.**
Figure S20: ^{13}C NMR (101 MHz) of compound **1j.**
Figure S21:^{1}H NMR (400 MHz) of compound **1k.**
Figure S22: ^{13}C NMR (101 MHz) of compound **1k.**
Figure S23:^{1}H NMR (400 MHz) of compound **1l.**
Figure S24: ^{13}C NMR (100 MHz) of compound **1l.**
Figure S25:^{1}H NMR (400 MHz) of compound **1m.**
Figure S26: ^{13}C NMR (101 MHz) of compound **1m.**
Figure S27:^{1}H NMR (400 MHz) of compound **1n.**
Figure S28: ^{13}C NMR (101 MHz) of compound **1n.**
Figure S29:^{1}H NMR (400 MHz) of compound **o.**
Figure S30: ^{13}C NMR (101 MHz) of compound **1o.**
Figure S31:^{1}H NMR (400 MHz) of compound **4d**
Figure S32: ^{13}C NMR (101 MHz) of compound **4d.**
Figure S33: *BOILED-Egg* diagram for compounds **1a-o**
Figure S34: radar plot calculated for compounds **1a-o**

Table S1. Elemental analysis of compounds **1** and **4d.** Compounds have been considered pure when the difference between calculated and found values is ± 0.4.

Comp.	Values	%C	%H	%N	%S
**1a**	Calcd.	54.24	5.36	14.88	8.52
	Found	54.00	5.21	15.69	8.22
**1b**	Calcd.	51.77	4.86	14.20	8.13
	Found	51.20	4.66	14.10	7.99
**1c**	Calcd.	51.77	4.86	14.20	8.13
	Found	51.52	5.00	14.32	8.00
**1d**	Calcd.	51.77	4.86	14.20	8.13
	Found	51.61	4.52	13.91	8.00
**1e**	Calcd.	55.37	5.68	14.35	8.21
	Found	55.63	5.69	14.46	8.31
**1f**	Calcd.	52.93	5.18	13.72	7.85
	Found	52.77	5.00	13.56	7.44
**1g**	Calcd.	52.93	5.18	13.72	7.85
	Found	52.87	5.10	13.56	7.56
**1h**	Calcd.	52.93	5.18	13.72	7.85
	Found	52.71	5.08	13.74	7.52
**1i**	Calcd.	56.42	5.98	13.85	7.93
	Found	56.60	5.95	13.98	8.10
**1j**	Calcd.	54.02	5.49	13.26	7.59
	Found	54.14	5.50	13.30	8.00
**1k**	Calcd.	54.02	5.49	13.26	7.59
	Found	54.00	5.40	13.10	7.80
**1l**	Calcd.	54.02	5.49	13.26	7.59
	Found	54.26	5.50	13.55	7.90
**1m**	Calcd.	57.40	6.26	13.39	7.66
	Found	57.69	6.83	13.56	7.13
**1n**	Calcd.	55.03	5.77	12.84	7.34
	Found	55.00	5.55	12.65	7.14
**1o**	Calcd.	55.03	5.77	12.84	7.34
	Found	55.02	5.65	12.84	6.33
**4d**	Calcd.	56.45	8.29	16.46	//
	Found	56.23	8.00	16.54	//

Figure S1:^{1}H NMR (400 MHz) of compound **1a**

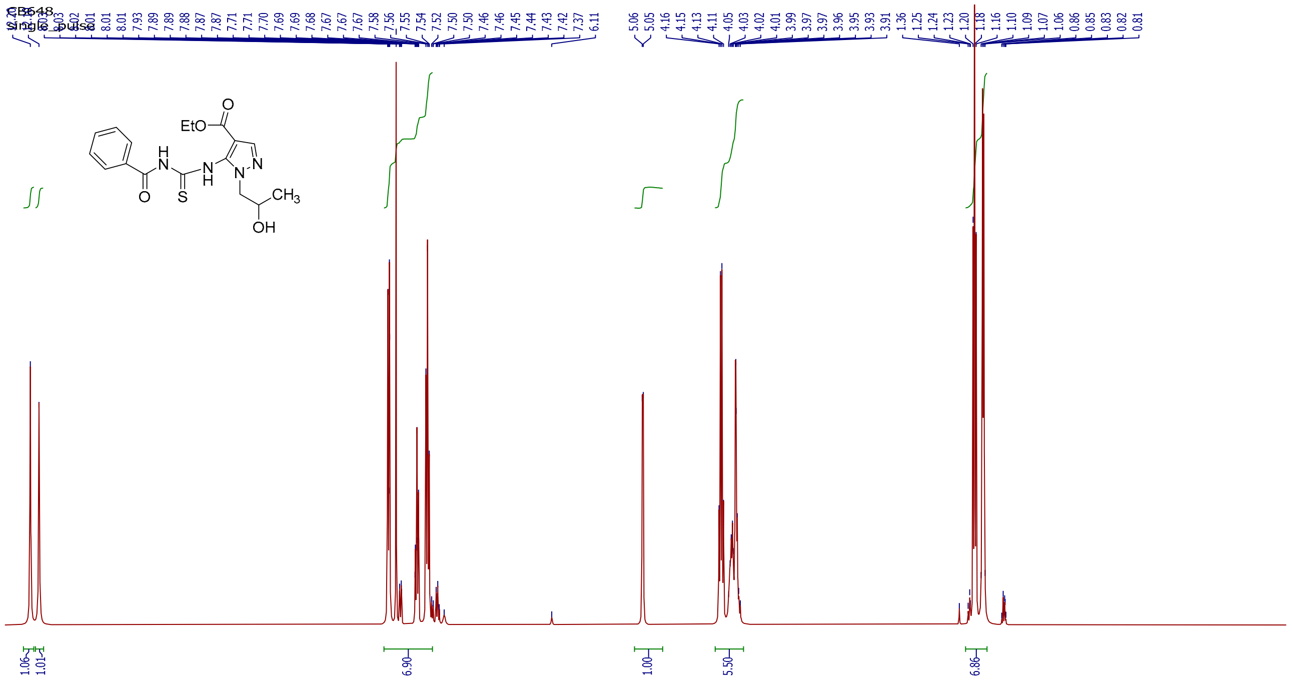

Figure S2: ^{13}C NMR (100 MHz) of compound **1a.**

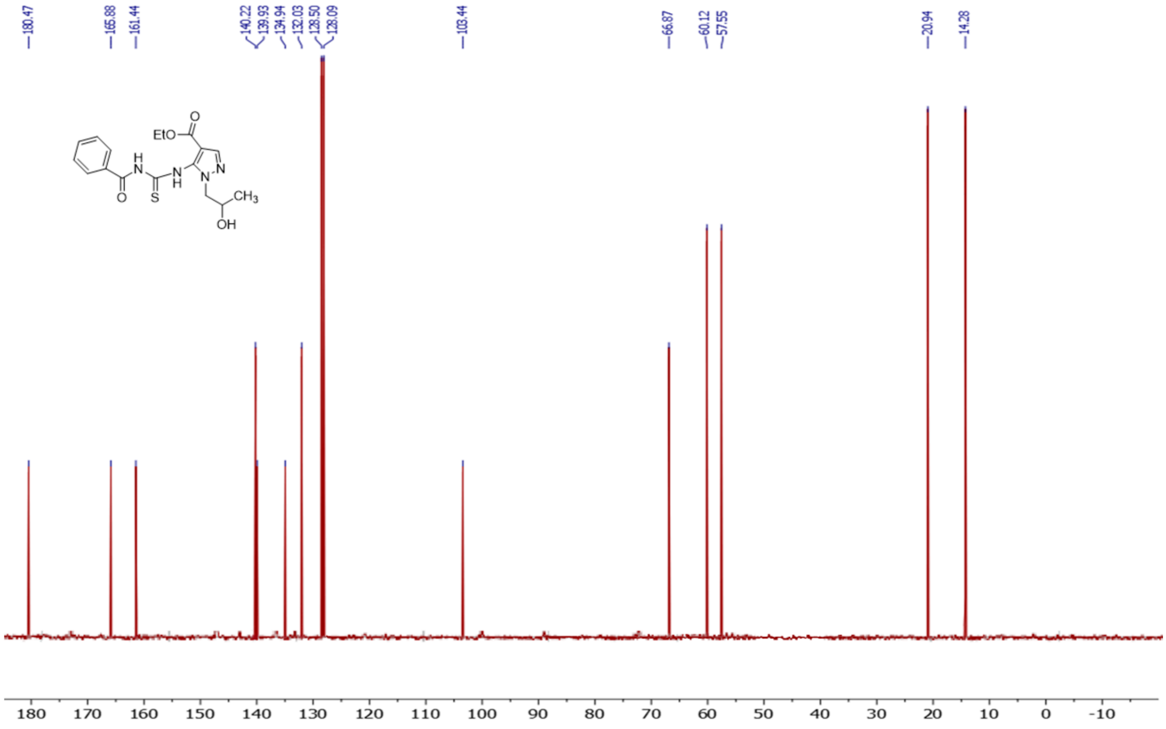

Figure S3: ^{1}H NMR (400 MHz) of compound **1b.**

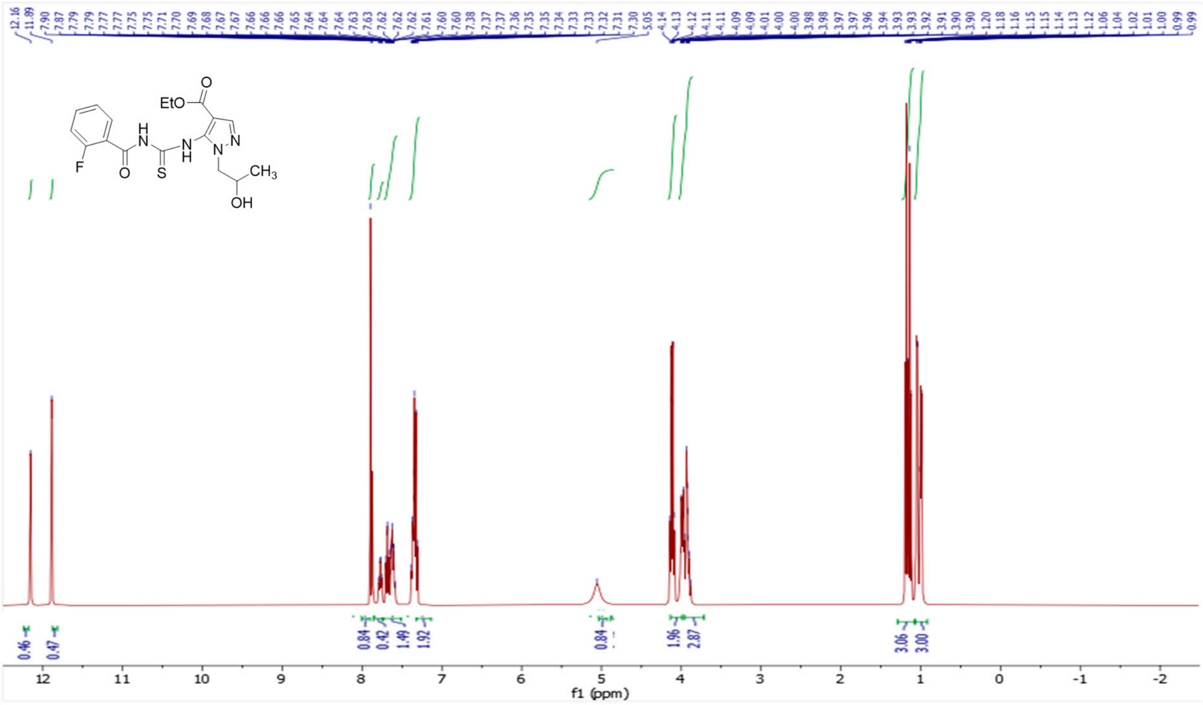

Figure S4: ^{13}C NMR (100 MHz) of compound **1b.**

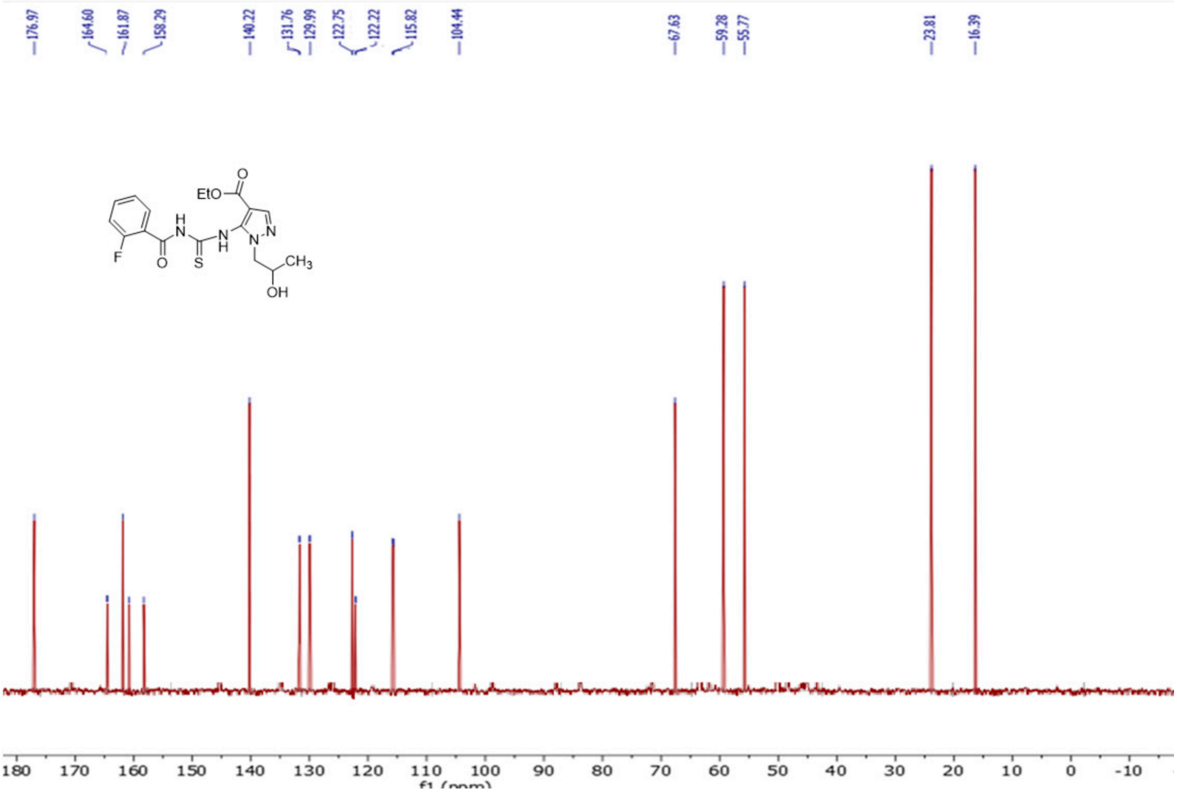

Figure S5:^{1}H NMR (400 MHz) of compound **1c.**

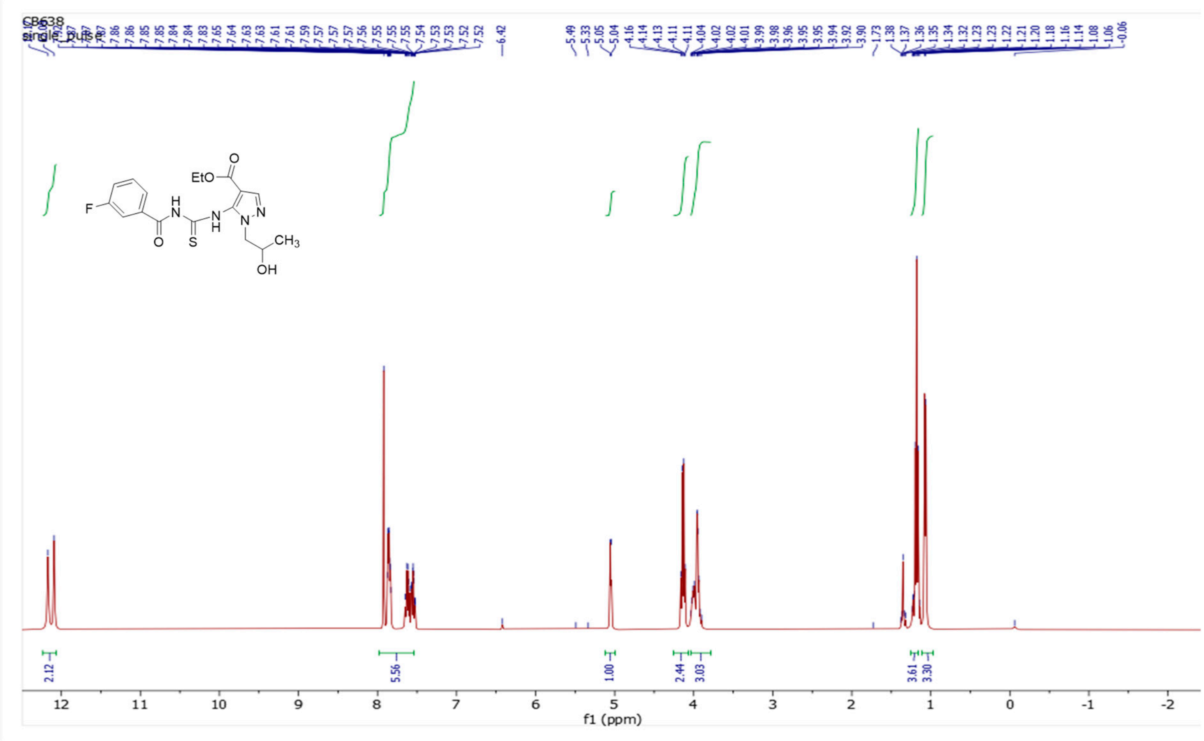

Figure S6: ^{13}C NMR (100 MHz) of compound **1c.**

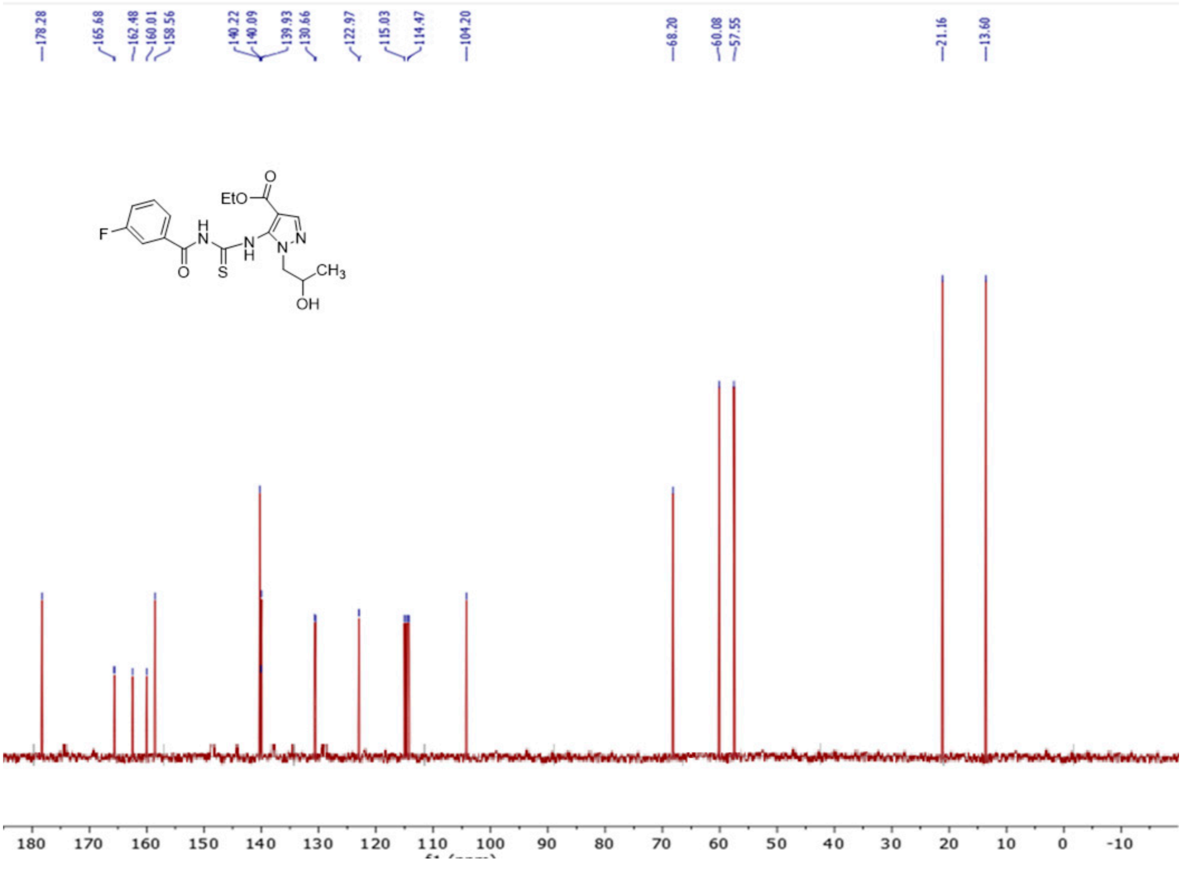

Figure S7:^{1}H NMR (400 MHz) of compound **1d.**

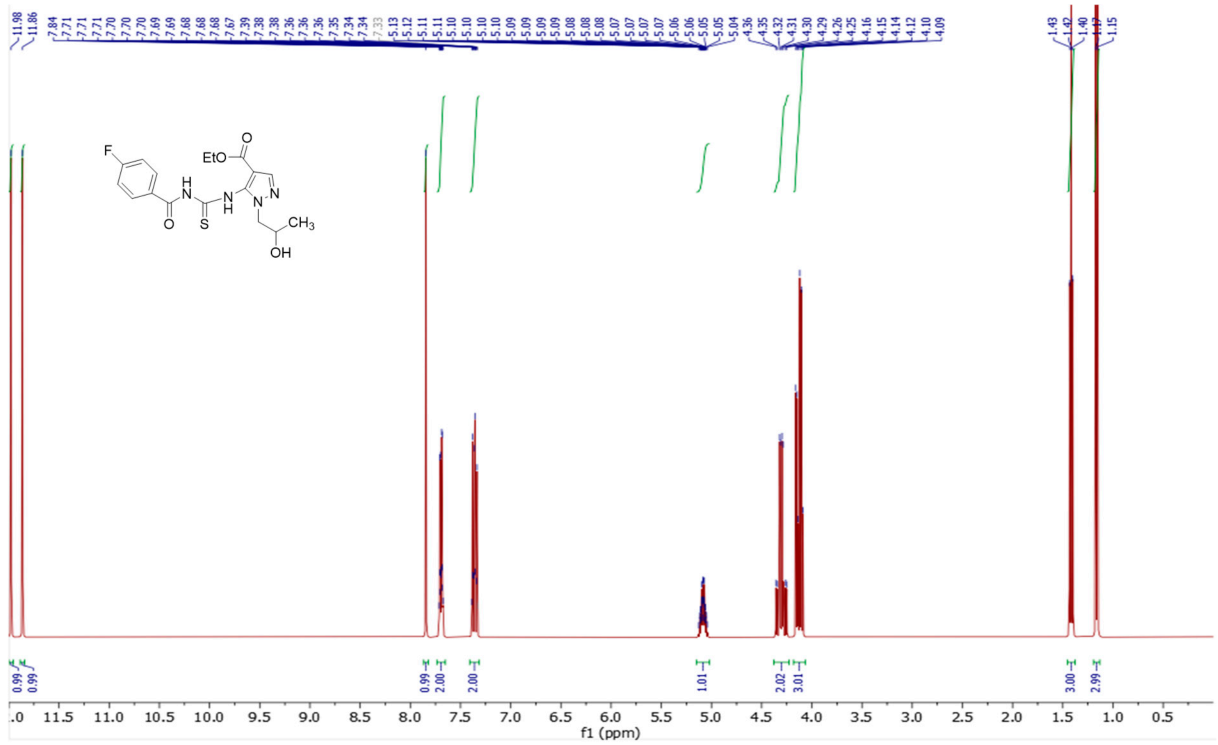

Figure S8: ^{13}C NMR (100 MHz) of compound **1d.**

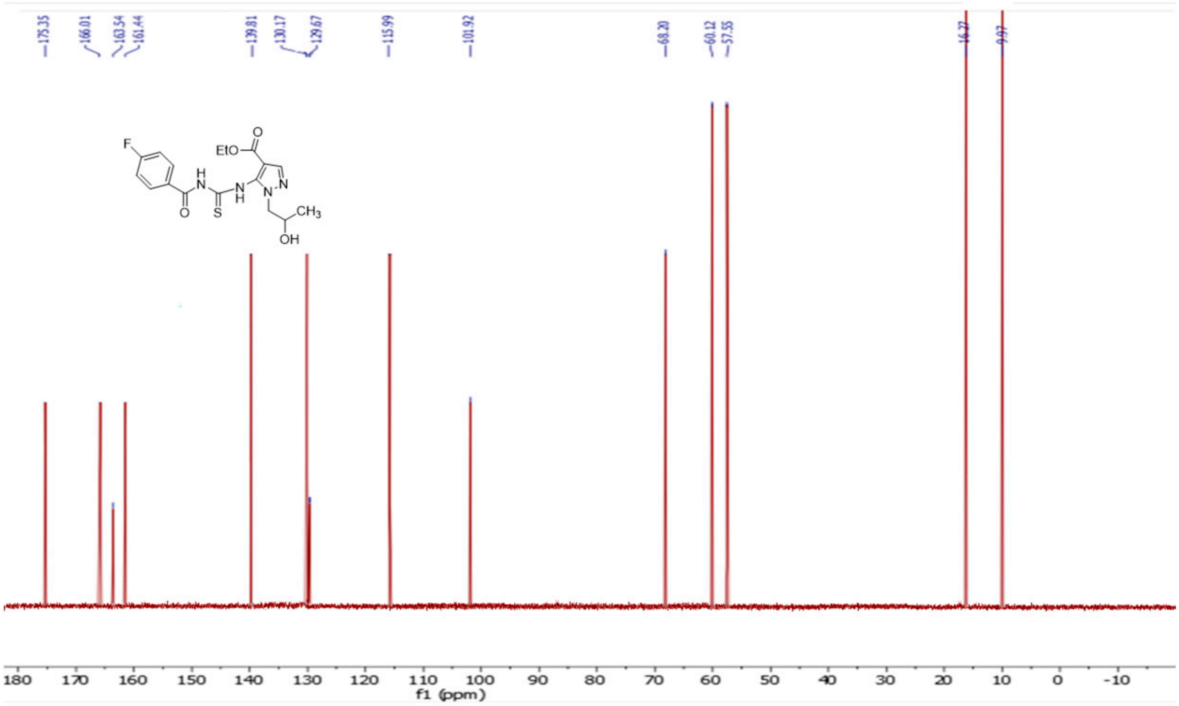

Figure S9:^{1}H NMR (400 MHz) of compound **1e.**

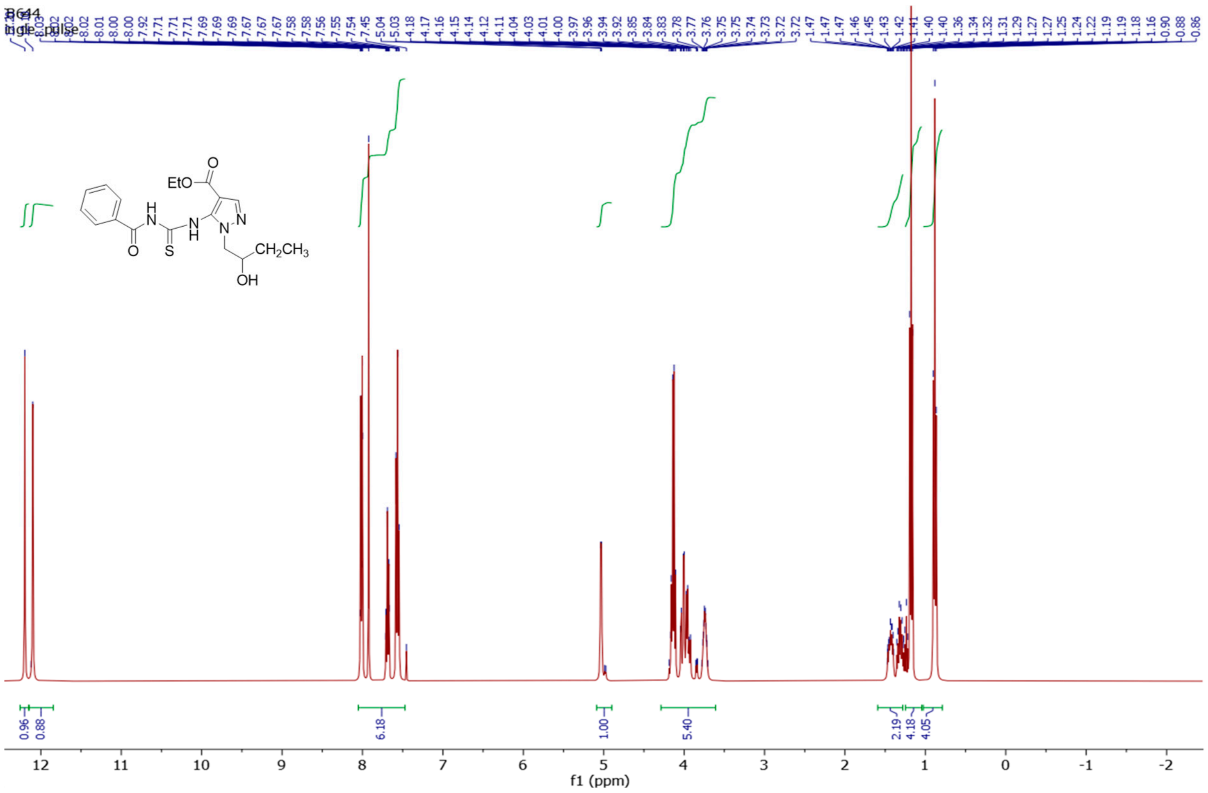

Figure S10: ^{13}C NMR (100 MHz) of compound **1e.**

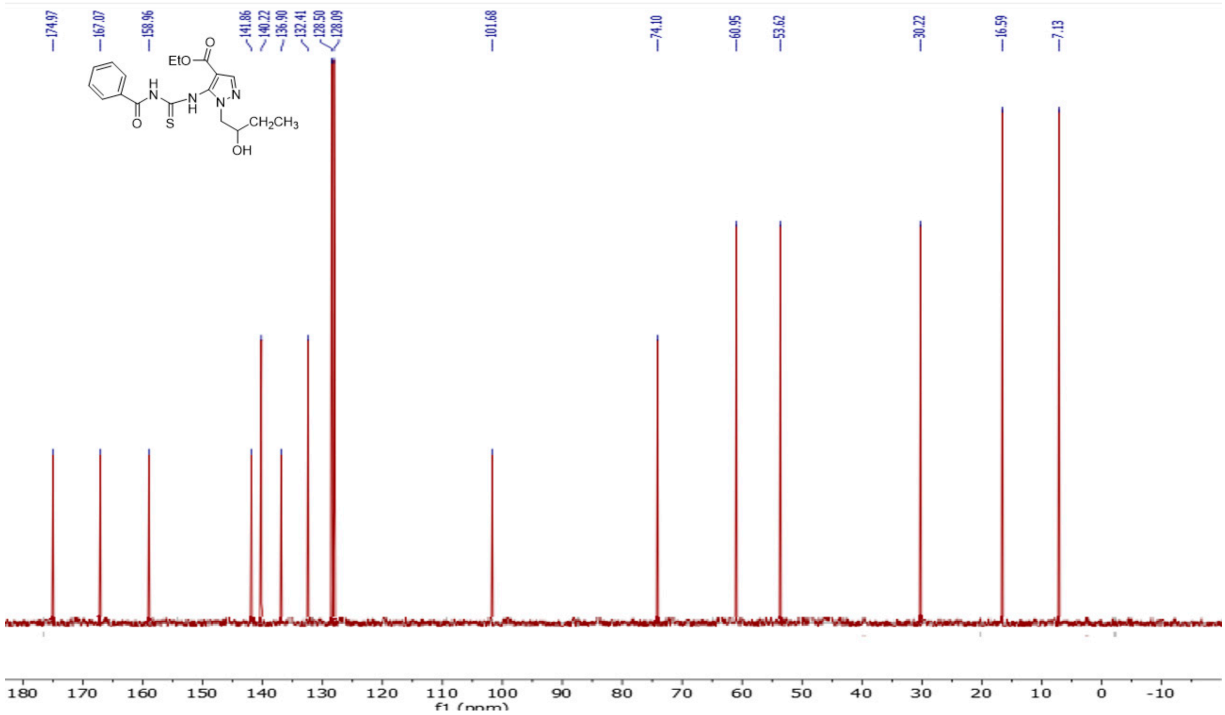

Figure S11:^{1}H NMR (400 MHz) of compound **1f.**

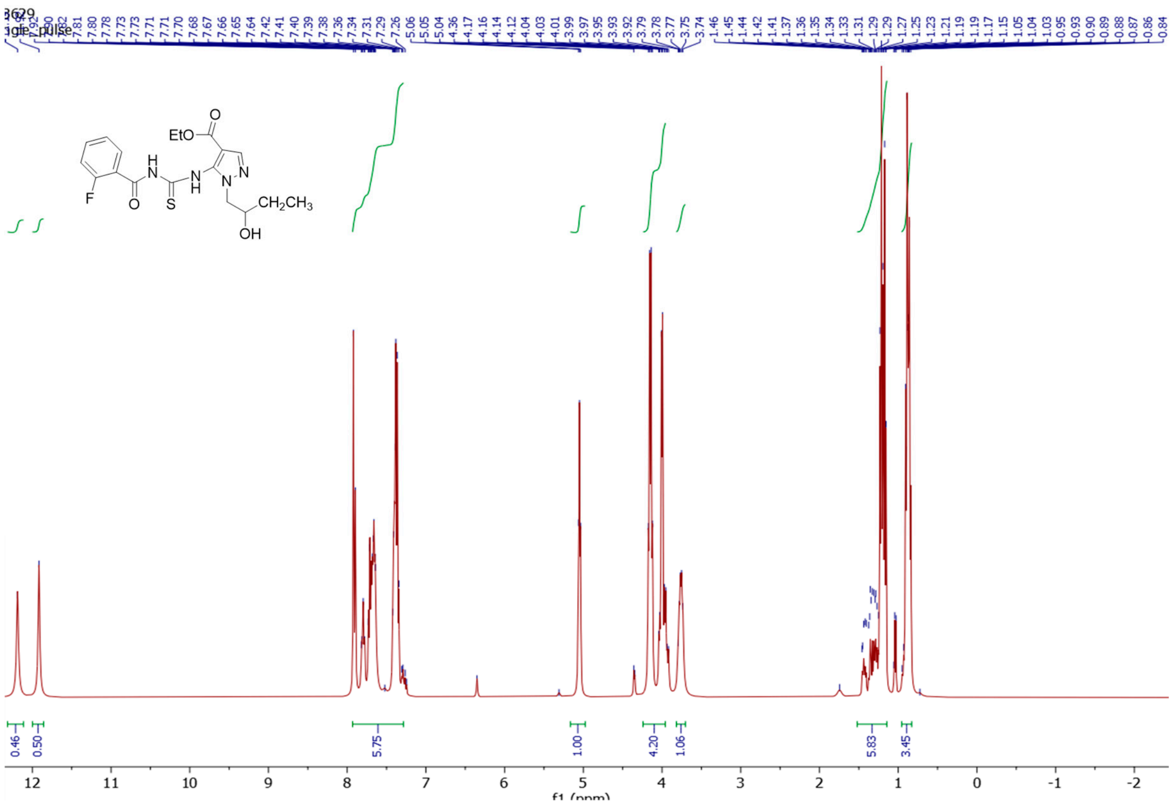

Figure S12: ^{13}C NMR (100 MHz) of compound **1f.**

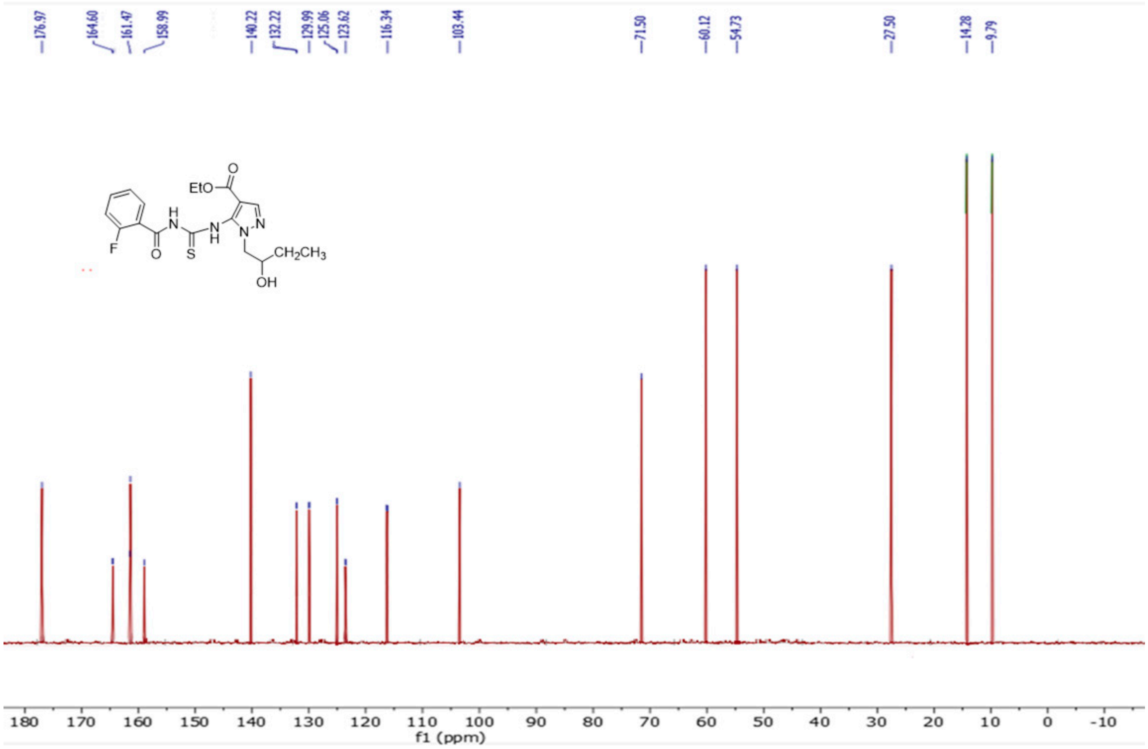

Figure S13:¹H NMR (400 MHz) of compound **1g.**

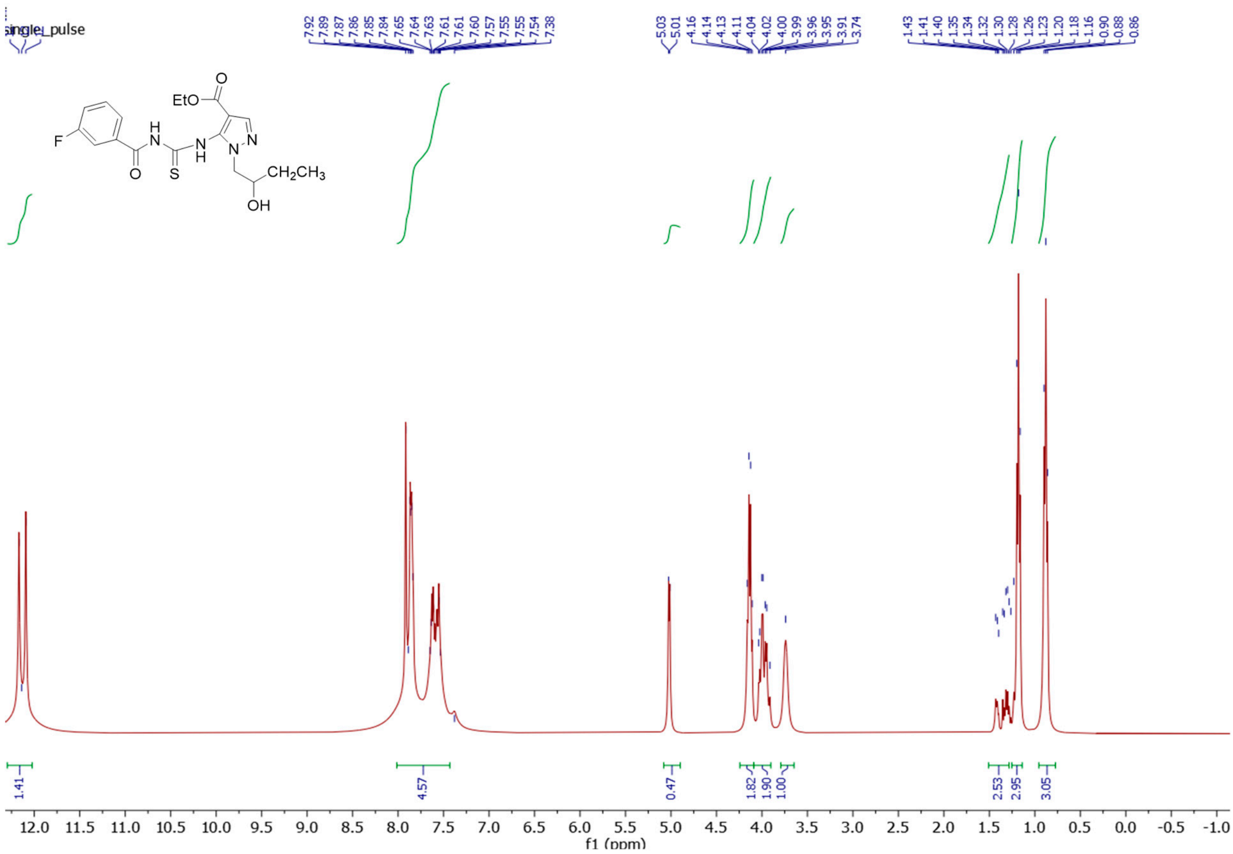

Figure S14: ¹³C NMR (100 MHz) of compound **1g.**

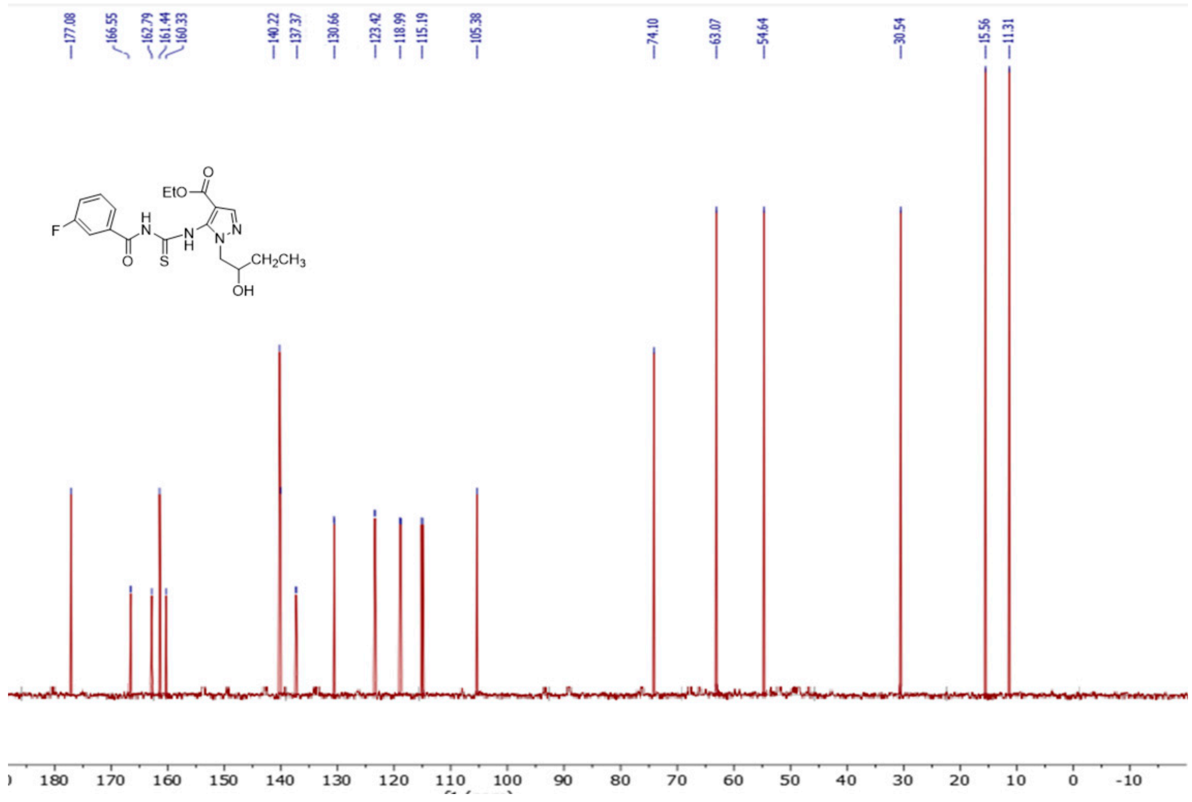

Figure S15:¹H NMR (400 MHz) of compound **1h.**

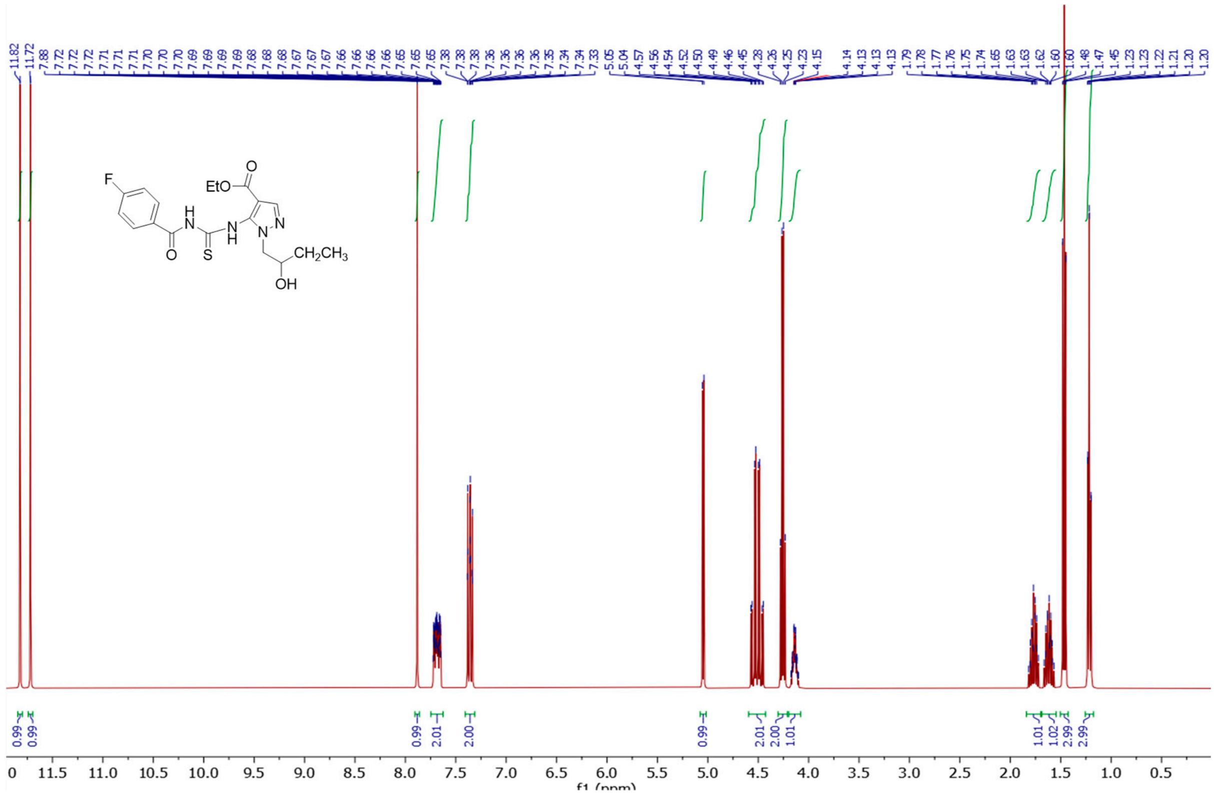

Figure S16: ¹³C NMR (100 MHz) of compound **1h.**

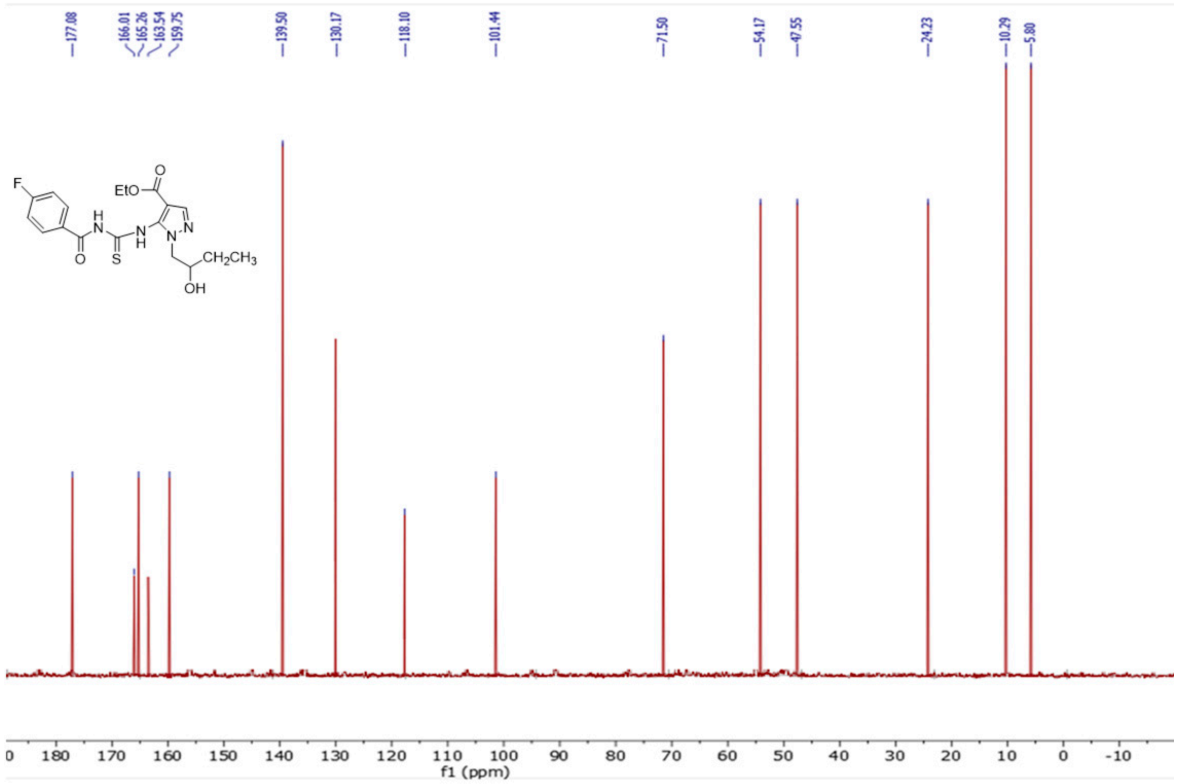

Figure S17:¹H NMR (400 MHz) of compound **1i.**

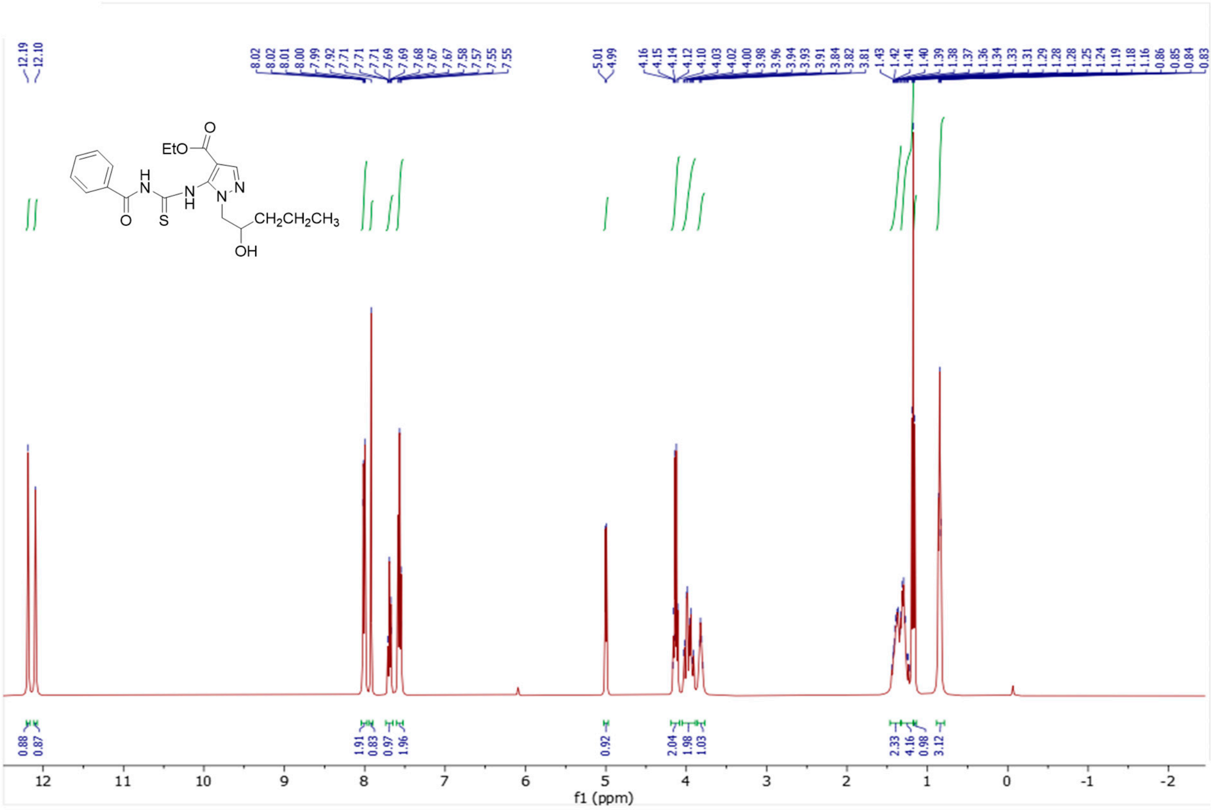

Figure S18: ¹³C NMR (100 MHz) of compound **1i.**

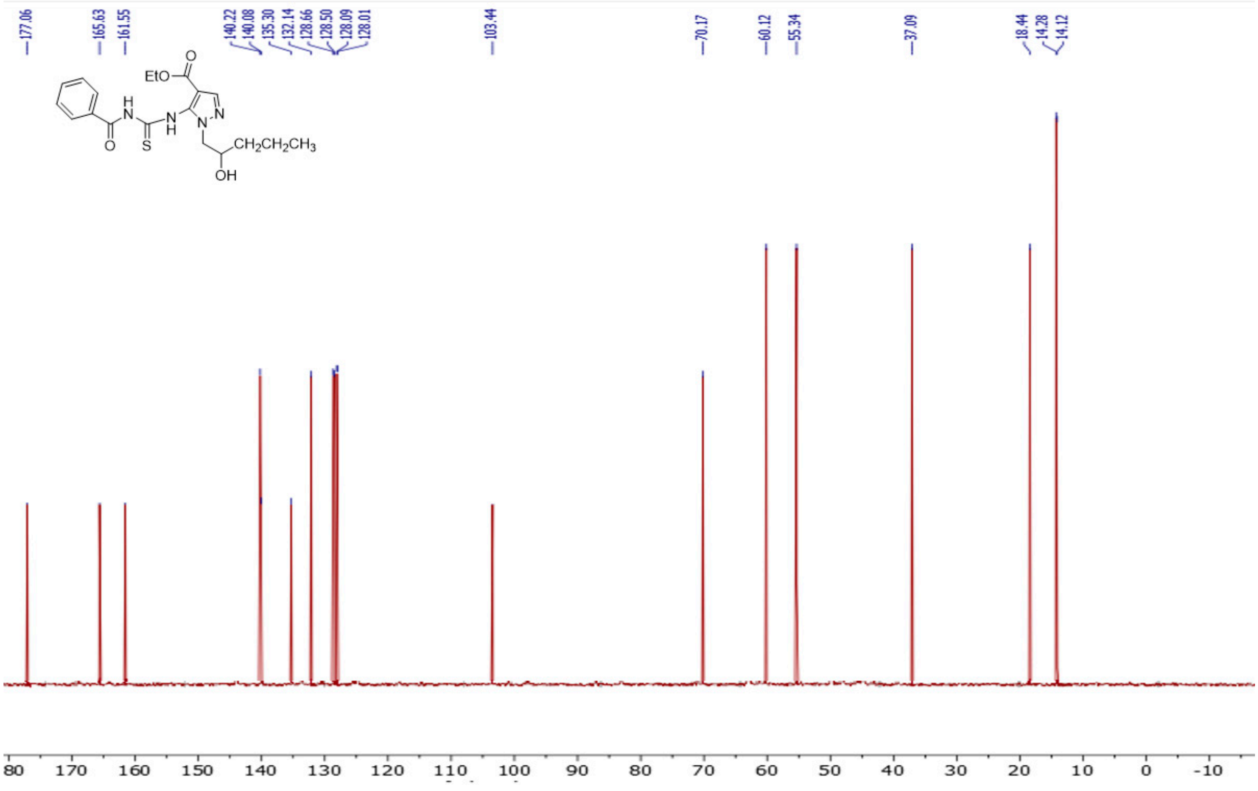

Figure S19:^{1}H NMR (400 MHz) of compound **1j.**

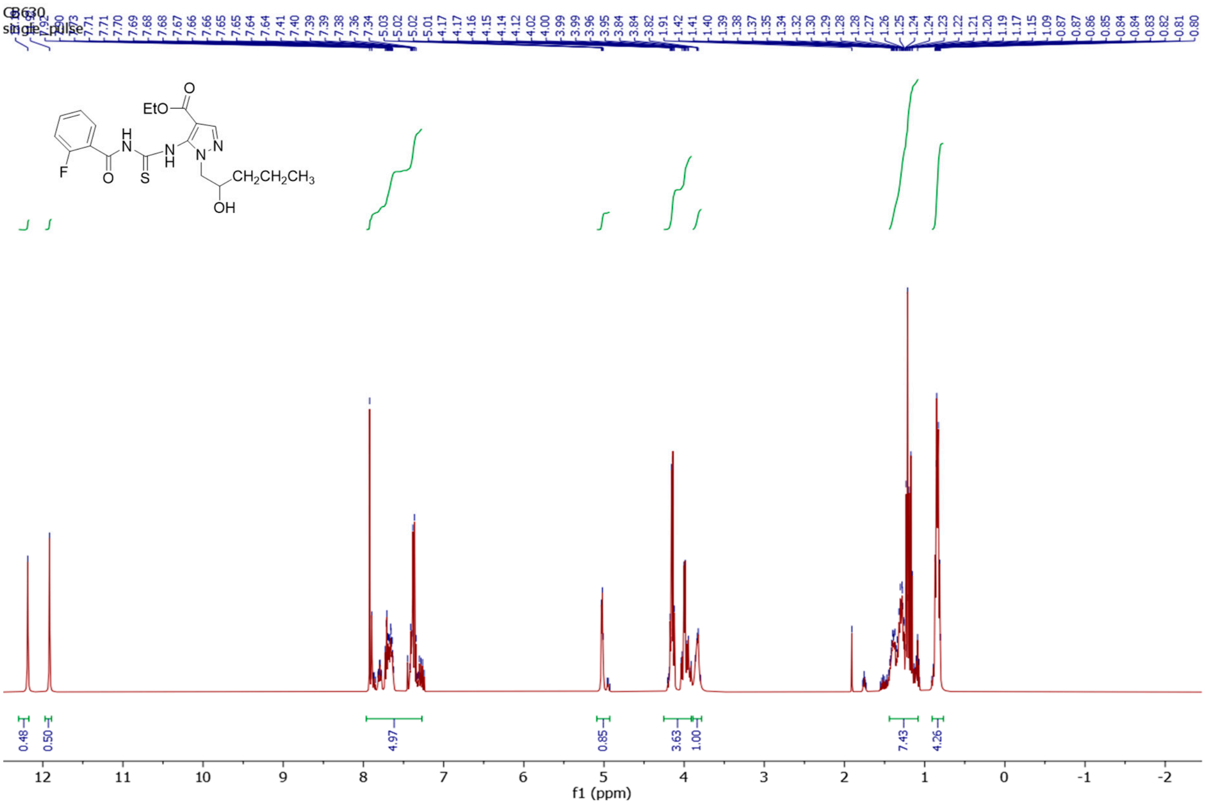

Figure S20: ^{13}C NMR (100 MHz) of compound **1j.**

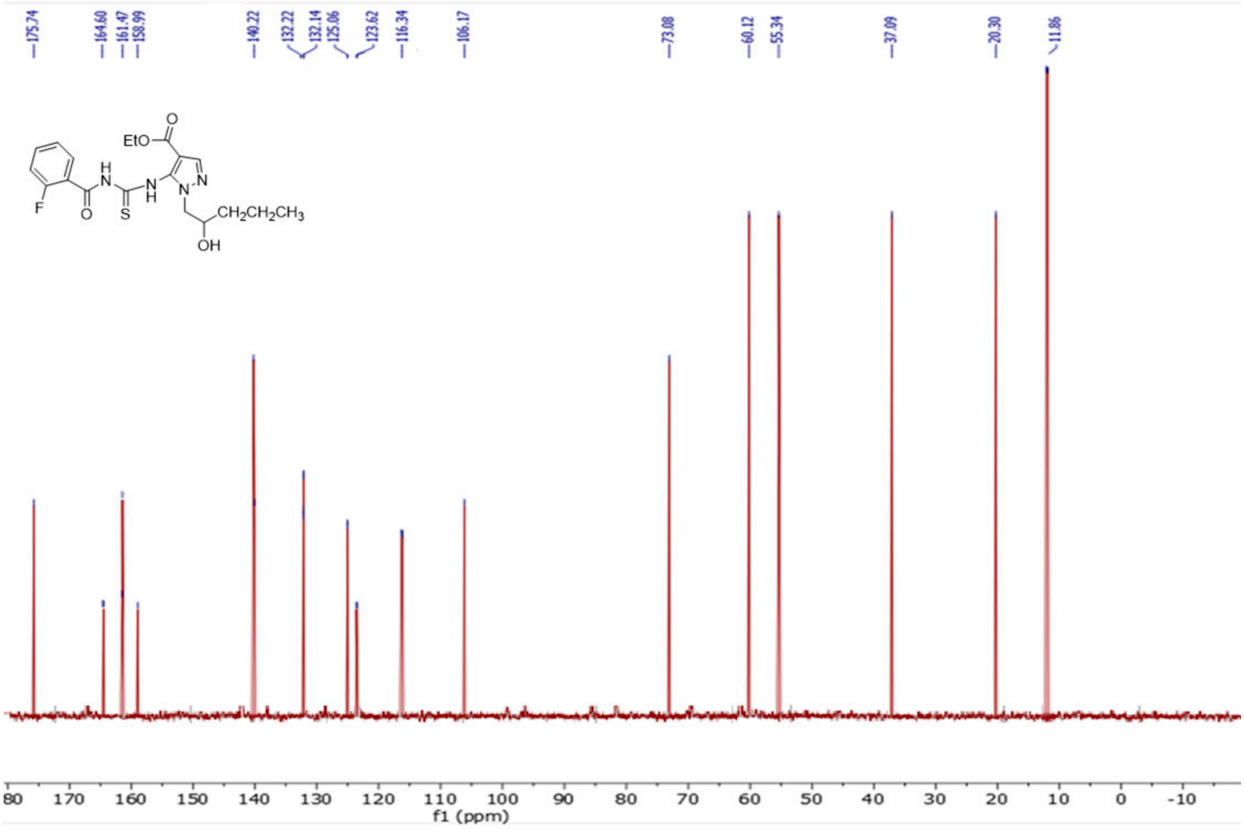

Figure S21: ¹H NMR (400 MHz) of compound **1k.**

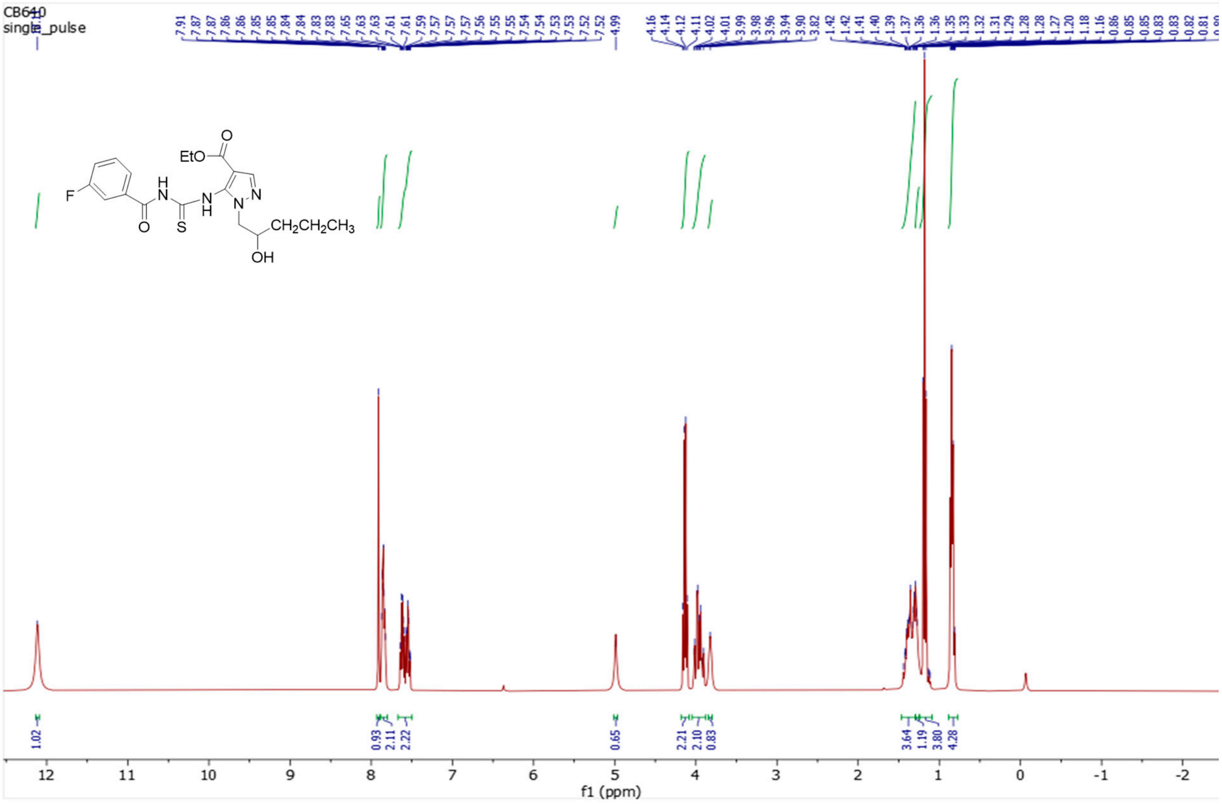

Figure S22: ¹³C NMR (100 MHz) of compound **1k.**

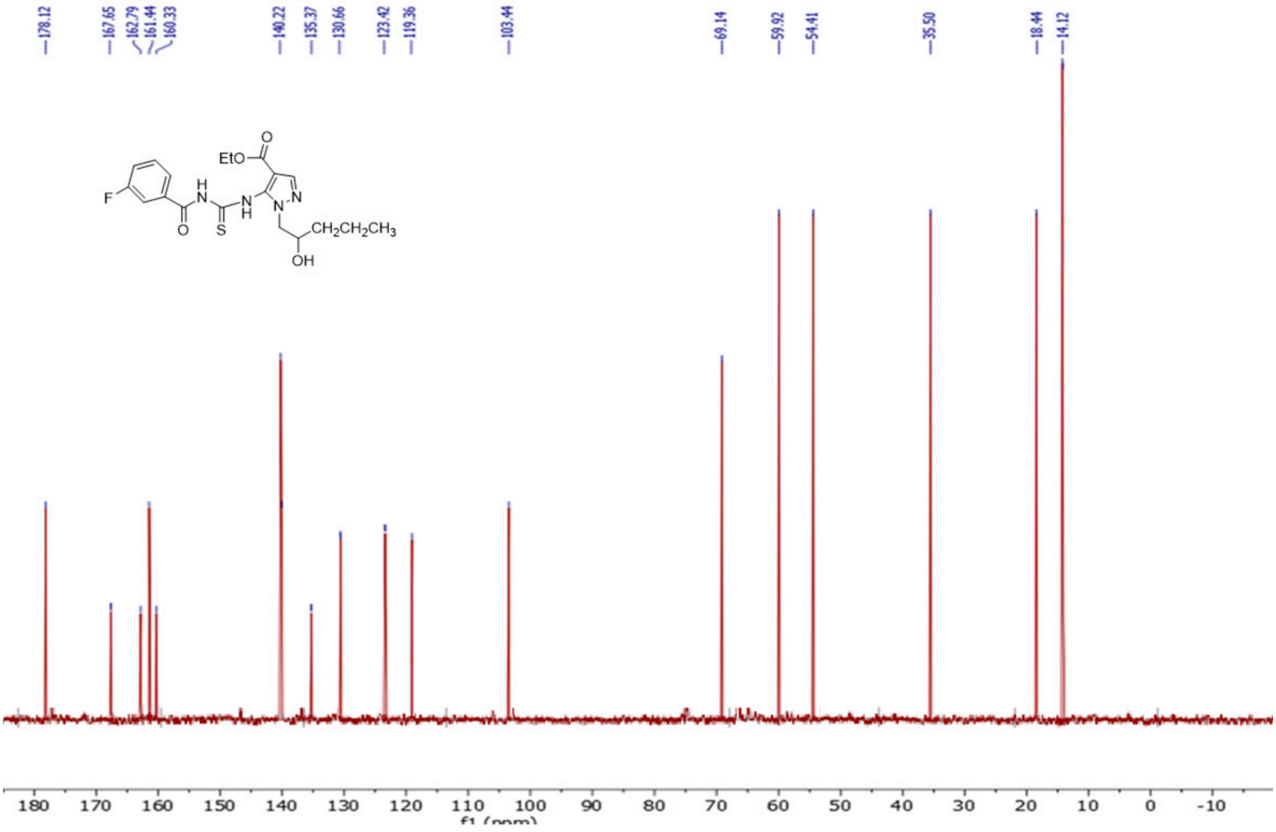

Figure S23:¹H NMR (400 MHz) of compound **1l.**

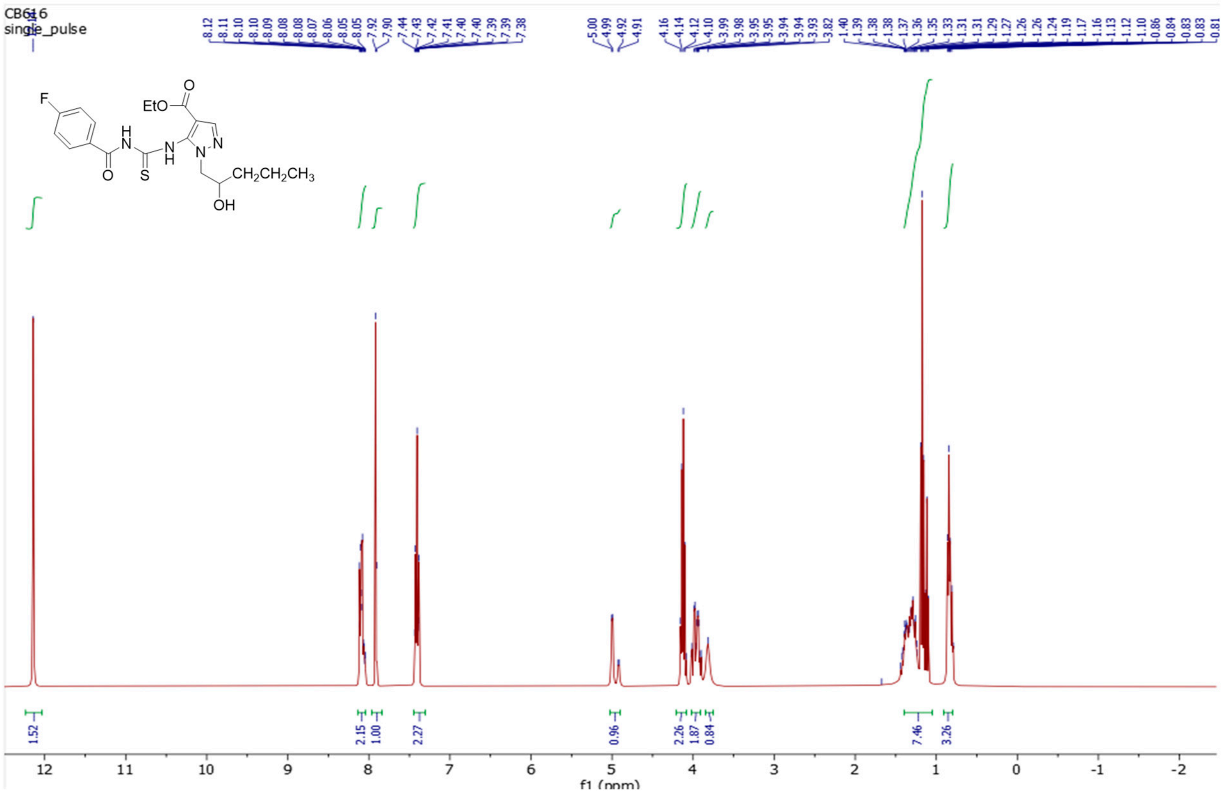

Figure S24: ¹³C NMR (100 MHz) of compound **1l.**

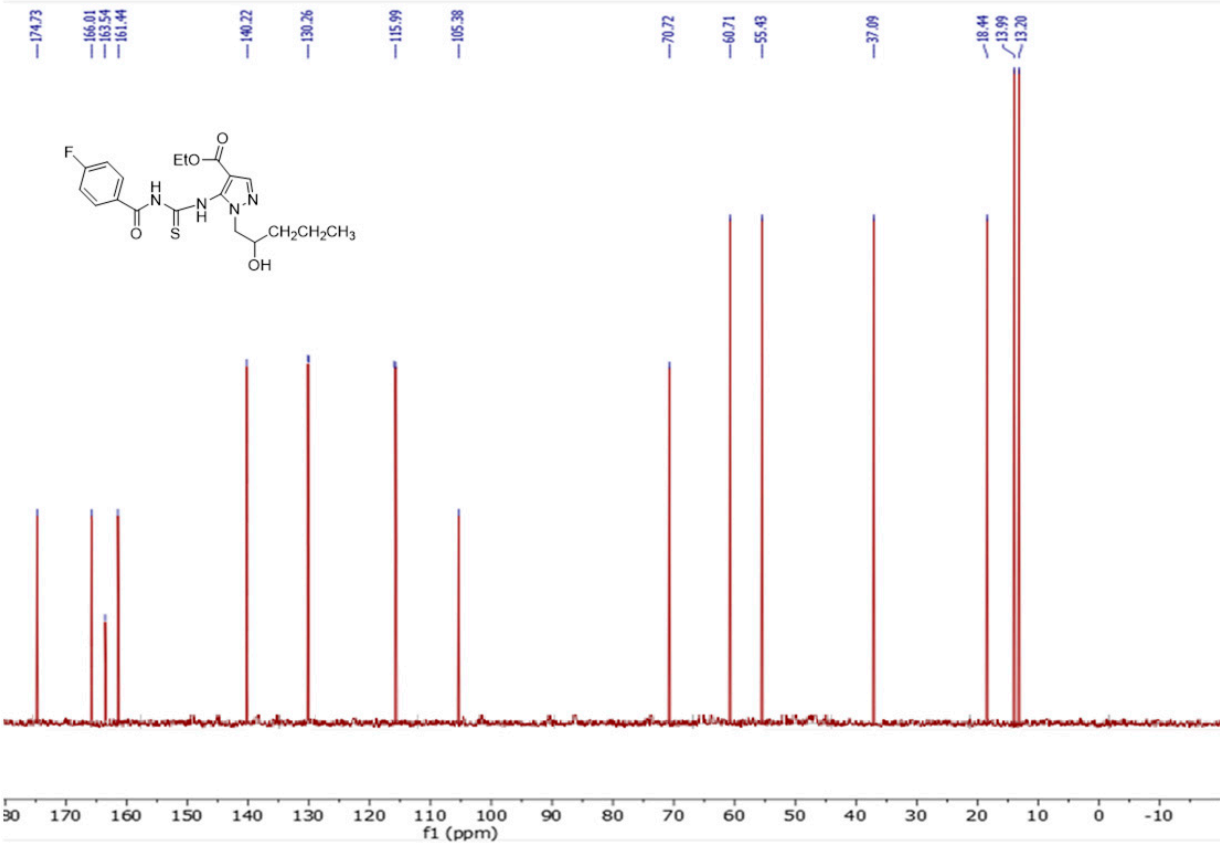

Figure S25:¹H NMR (400 MHz) of compound **1m.**

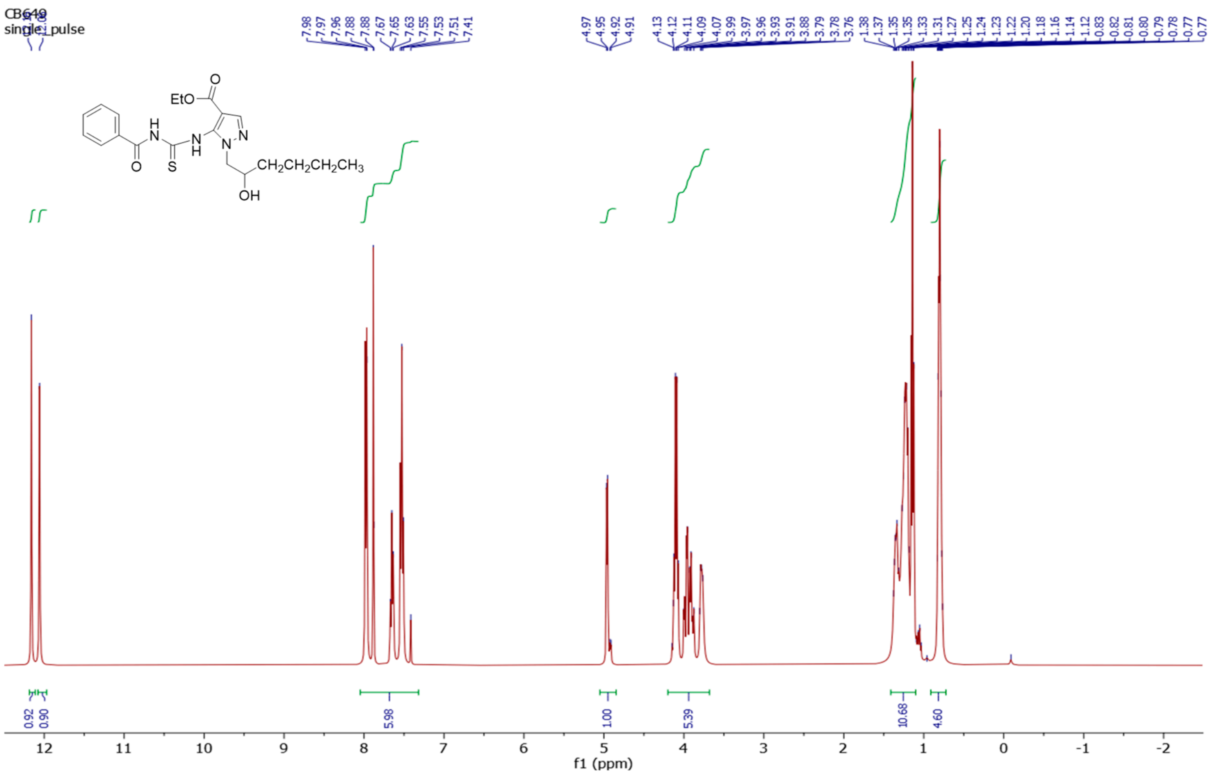

Figure S26: ¹³C NMR (100 MHz) of compound **1m.**

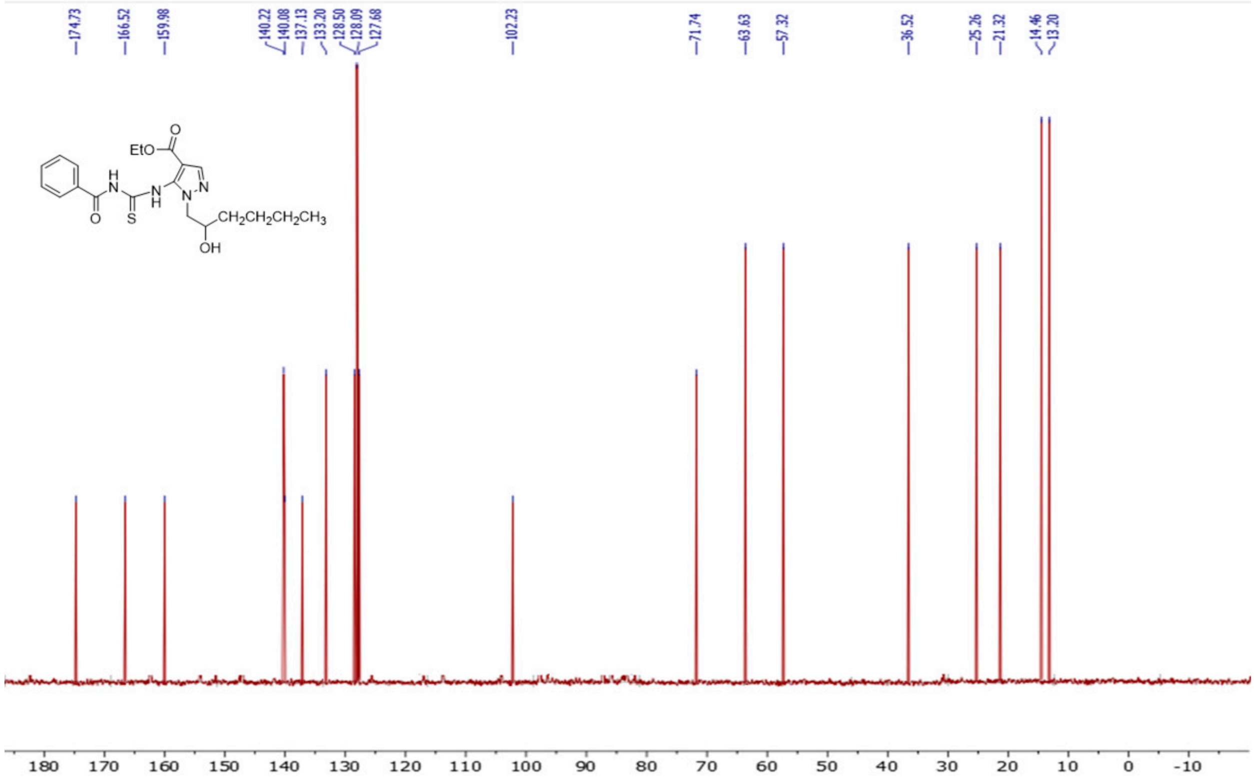

Figure S27:^{1}H NMR (400 MHz) of compound **1n.**

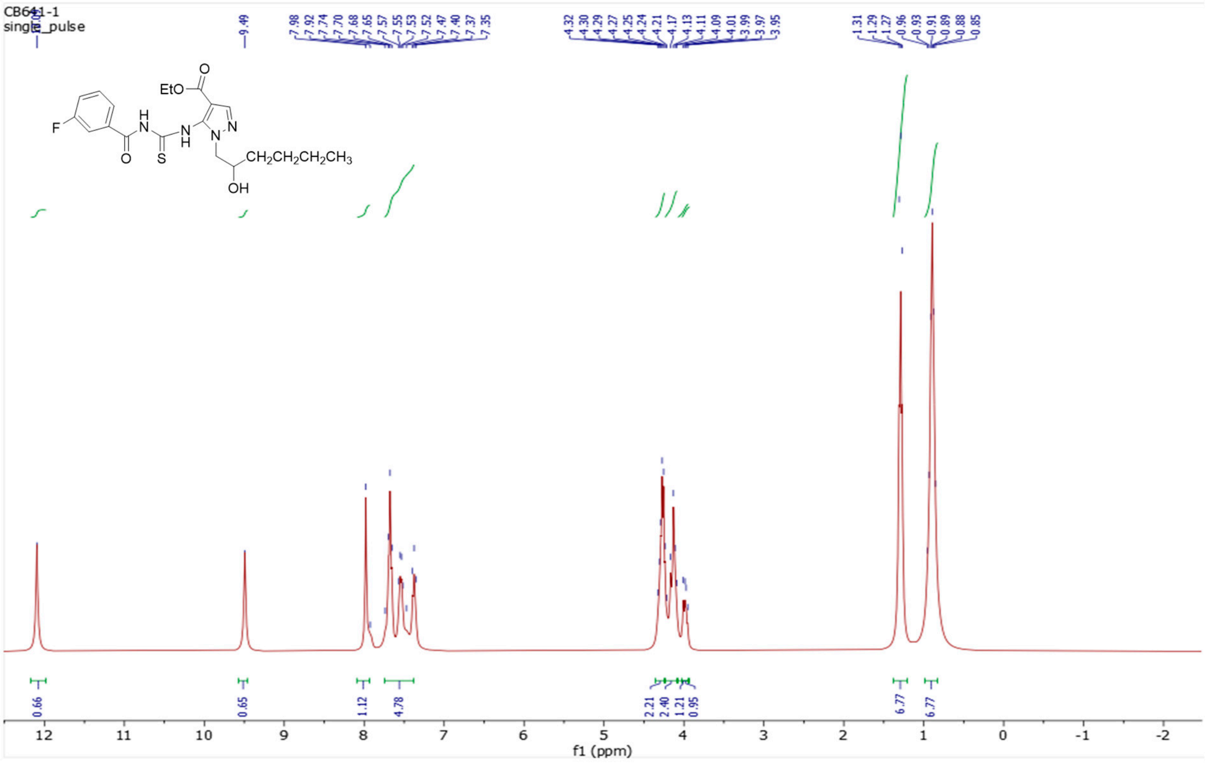

Figure S28: ^{13}C NMR (100 MHz) of compound **1n.**

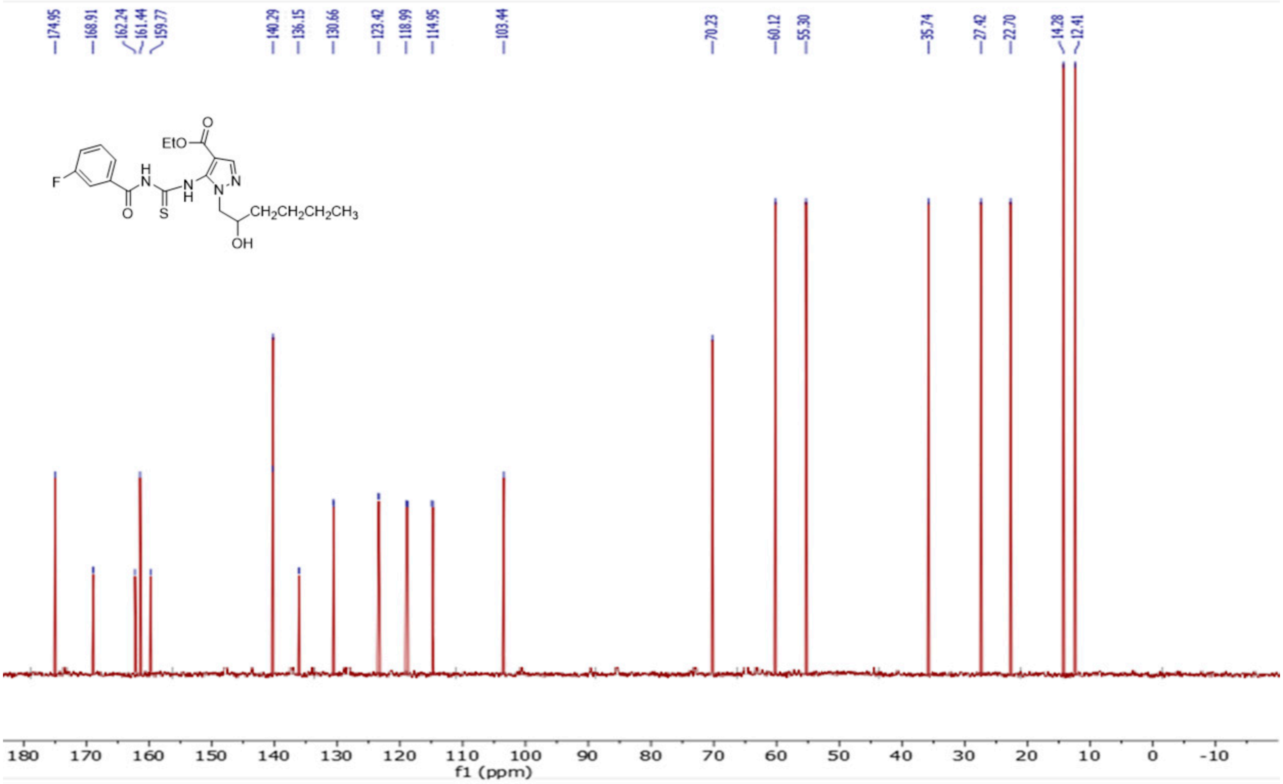

Figure S29:^{1}H NMR (400 MHz) of compound **1o.**

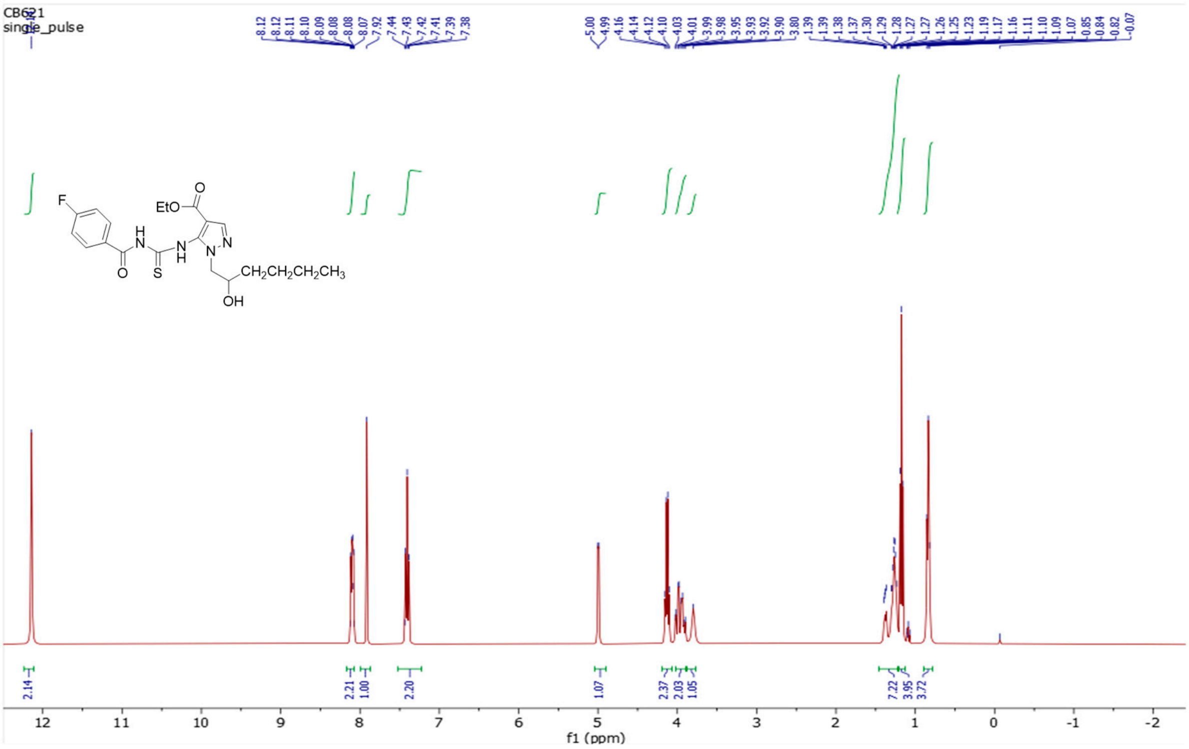

Figure S30: ^{13}C NMR (100 MHz) of compound **1o.**

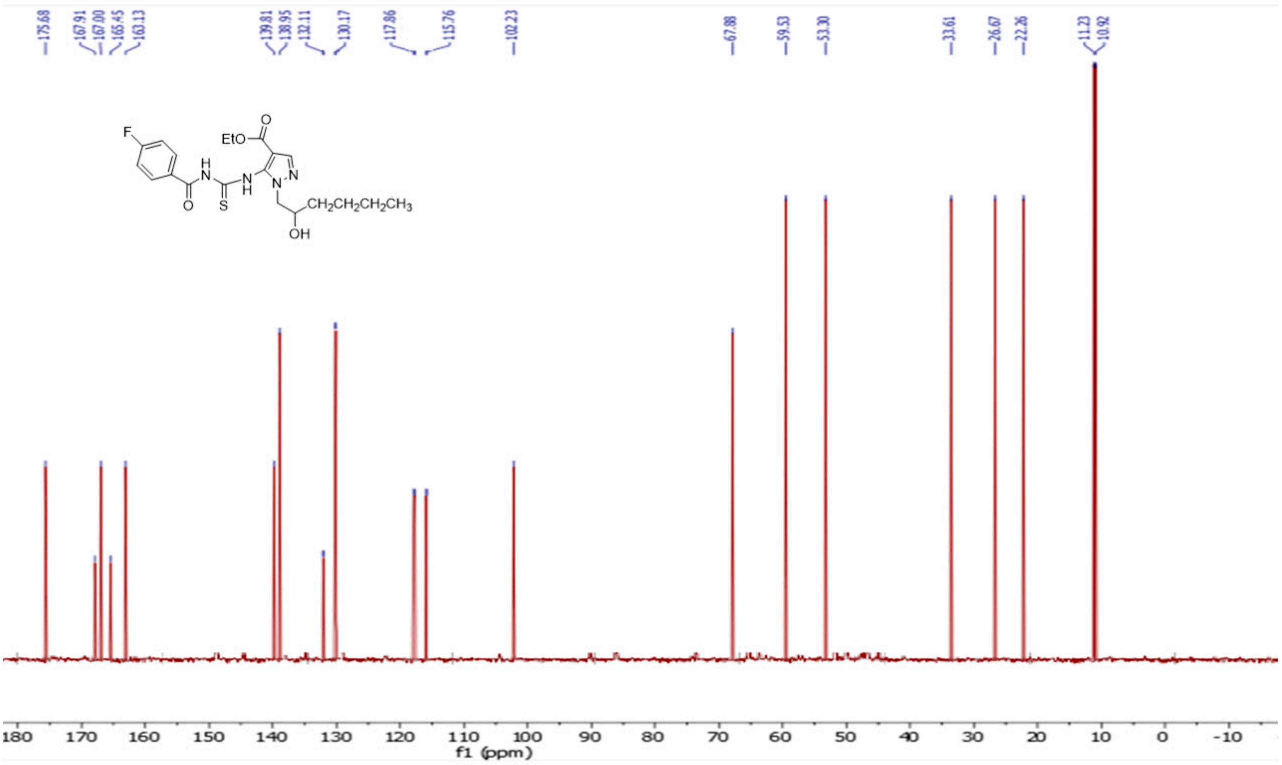

Figure S31: ¹H NMR (400 MHz) of compound **4d.**

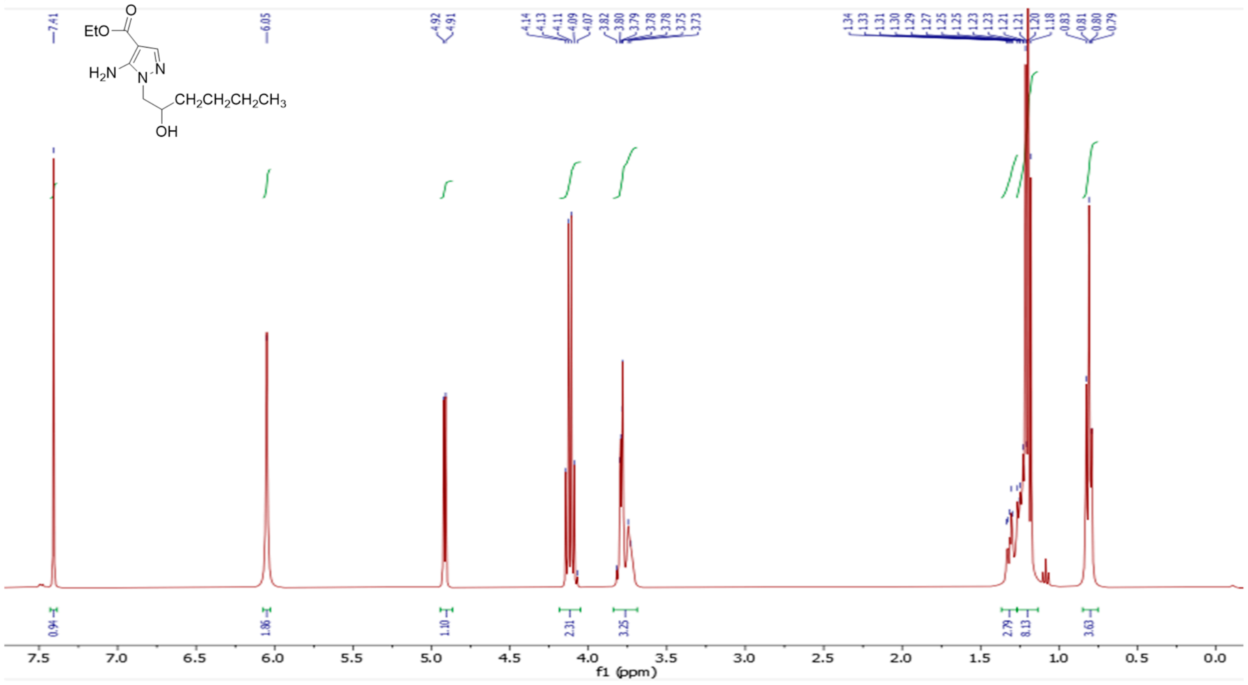

Figure S32: ¹³C NMR (100 MHz) of compound **4d.**

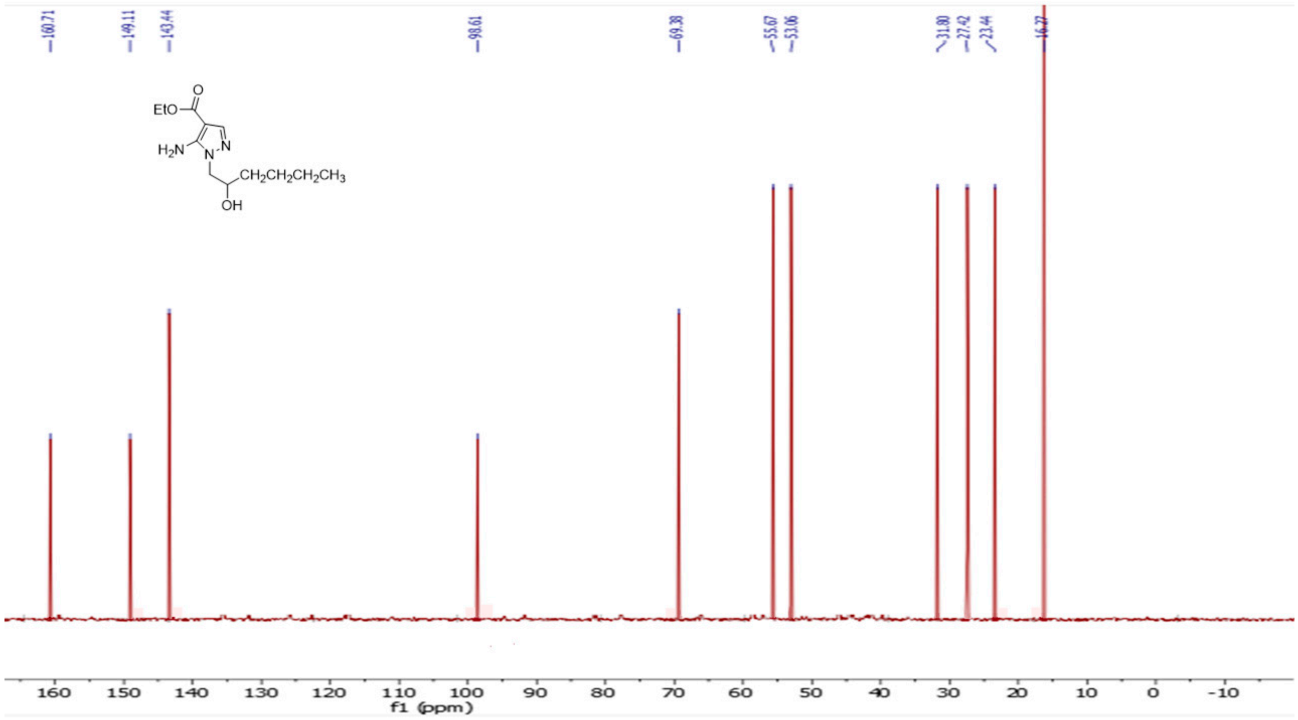

Figure S33: *BOILED-Egg* diagram for compounds **1a-o**

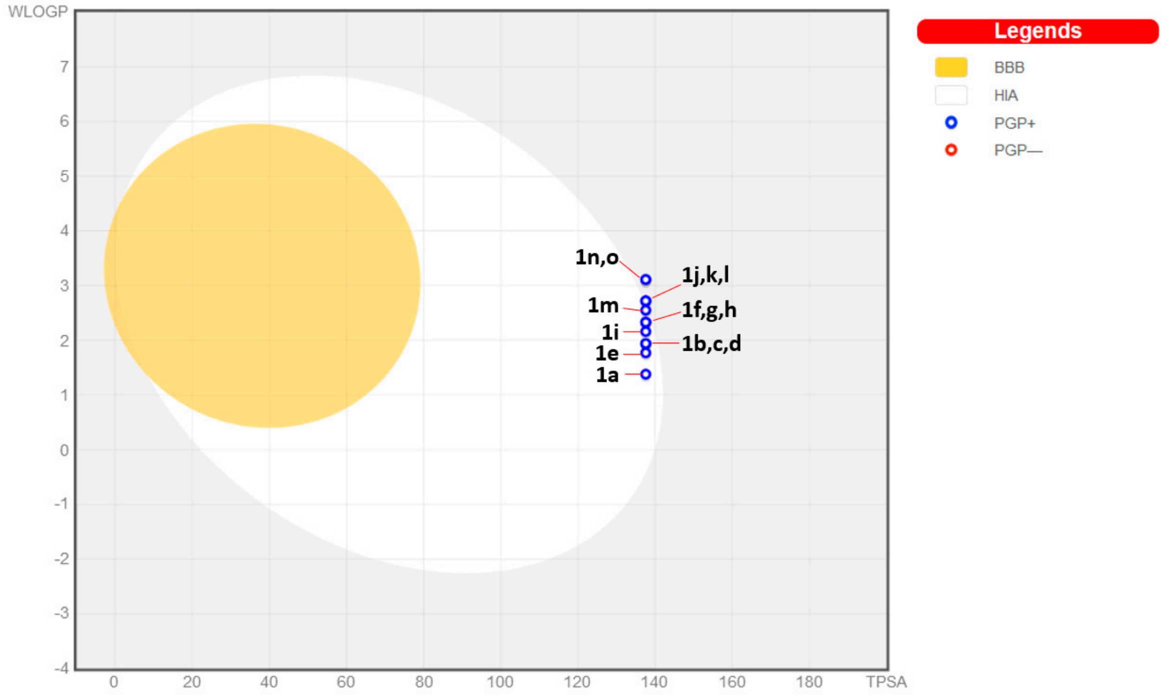

Figure S34: radar plot calculated for compounds **1a-o**

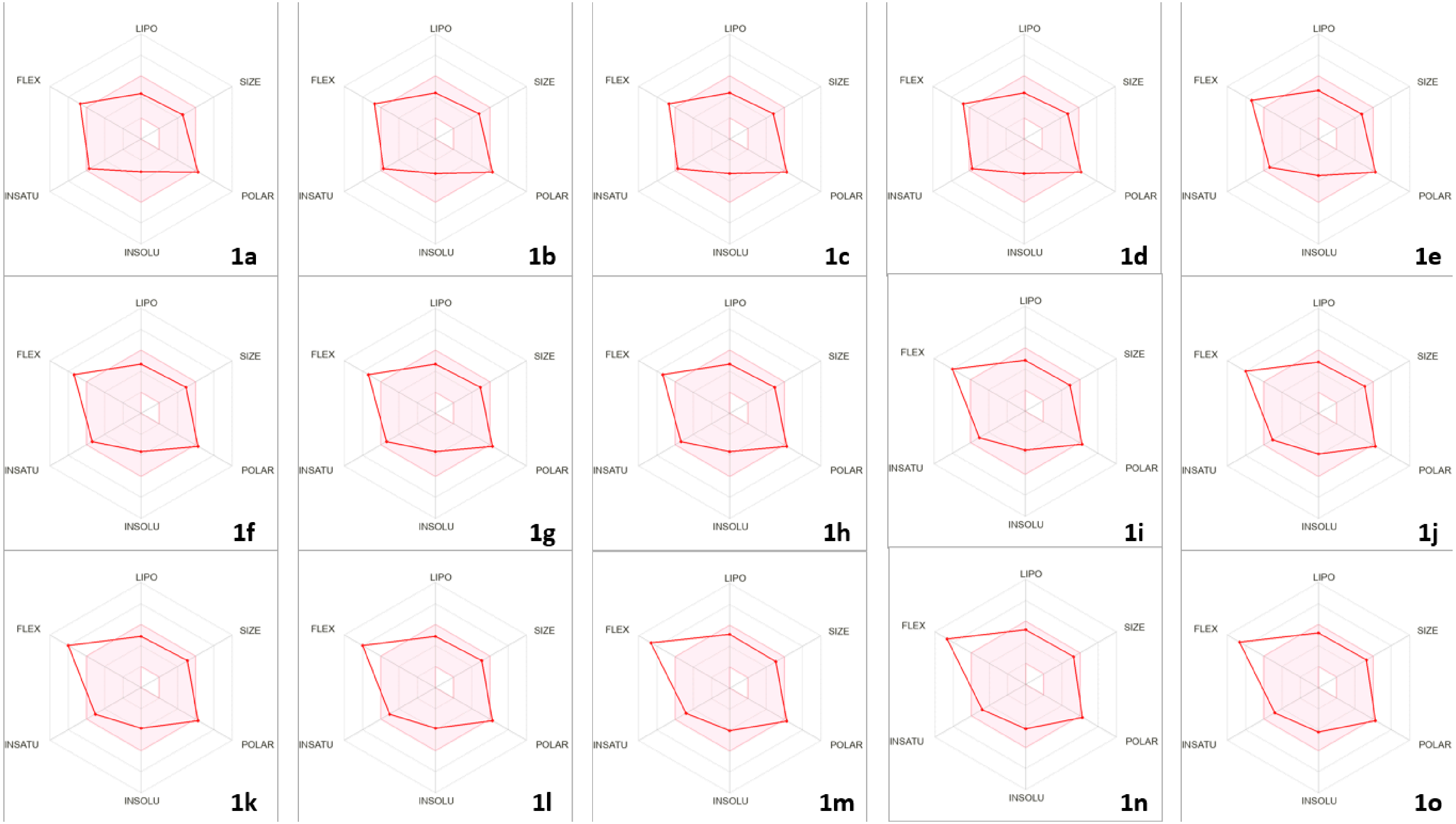

Supplement: Supplementary file 1 [file pharmaceuticals-17-00376-s001.zip › pharmaceuticals-2768454-supplementary.pdf]
